# Hydrodynamic cavitation for prebiotic ribose formation from H_2_O and CO_2_

**DOI:** 10.1093/nsr/nwag182

**Published:** 2026-03-23

**Authors:** Yuxi Fang, Yicong Luo, Wei Ding, Diwen Ying, Wanning Zhang, Jiaxin Song, Wanbin Zhang, Jinping Jia

**Affiliations:** State Key Laboratory of Synergistic Chem-Bio Synthesis, School of Chemistry and Chemical Engineering, Frontiers Science Center for Transformative Molecules, Shanghai Key Laboratory for Molecular Engineering of Chiral Drugs, Shanghai Jiao Tong University, Shanghai 200240, China; State Key Laboratory of Synergistic Chem-Bio Synthesis, School of Chemistry and Chemical Engineering, Frontiers Science Center for Transformative Molecules, Shanghai Key Laboratory for Molecular Engineering of Chiral Drugs, Shanghai Jiao Tong University, Shanghai 200240, China; School of Environmental Science and Engineering, Shanghai Jiao Tong University, Shanghai 200240, China; School of Environmental Science and Engineering, Shanghai Jiao Tong University, Shanghai 200240, China; State Key Laboratory of Synergistic Chem-Bio Synthesis, School of Chemistry and Chemical Engineering, Frontiers Science Center for Transformative Molecules, Shanghai Key Laboratory for Molecular Engineering of Chiral Drugs, Shanghai Jiao Tong University, Shanghai 200240, China; State Key Laboratory of Synergistic Chem-Bio Synthesis, School of Chemistry and Chemical Engineering, Frontiers Science Center for Transformative Molecules, Shanghai Key Laboratory for Molecular Engineering of Chiral Drugs, Shanghai Jiao Tong University, Shanghai 200240, China; State Key Laboratory of Synergistic Chem-Bio Synthesis, School of Chemistry and Chemical Engineering, Frontiers Science Center for Transformative Molecules, Shanghai Key Laboratory for Molecular Engineering of Chiral Drugs, Shanghai Jiao Tong University, Shanghai 200240, China; State Key Laboratory of Synergistic Chem-Bio Synthesis, School of Chemistry and Chemical Engineering, Frontiers Science Center for Transformative Molecules, Shanghai Key Laboratory for Molecular Engineering of Chiral Drugs, Shanghai Jiao Tong University, Shanghai 200240, China; School of Environmental Science and Engineering, Shanghai Jiao Tong University, Shanghai 200240, China

**Keywords:** origin of life, prebiotic synthesis, CO_2_ reduction, cavitation, ribose

## Abstract

H_2_O and CO_2_ are considered as fundamental precursors for the prebiotic formation of biomolecules. Their conversion to biomolecules under early Earth conditions is critical for understanding origin of life. However, direct formation from these simple compounds remains unverified, leaving the mechanistic role of H_2_O unresolved. Herein, we demonstrate ribose synthesis from H_2_O and CO_2_ under prebiotic conditions *via* hydrodynamic cavitation, a process naturally occurring in ancient oceans. Ca^2+^-mediated hydrodynamic cavitation in a Venturi reactor produced ribose as the major sugar product. Ribose was predicted to be formed from formaldehyde and CH_2_OH· radicals generated *via* a sequential reaction involving OH·, H·, and C_1_· radicals derived from H_2_O and CO_2_ under high-temperature/pressure induced by hydrodynamic cavitation, followed by successive CH_2_OH· radical substitutions. These findings indicate that early Earth’s aqueous fluids could enable abiotic biomolecule formation, suggesting flowing H_2_O acted as both driver and reactant in life’s chemical origin.

## Teaser

Hydrodynamic cavitation was observed to directly convert carbon dioxide and water into ribose, demonstrating that fluids on early Earth might have naturally produced life’s building blocks.

## INTRODUCTION

Hydrodynamic cavitation is prevalent in natural water body [1–4]. Hydrodynamic cavitation, a fluid dynamics phenomenon, produces extremely high temperatures and pressures through instantaneous bubble collapse [5–7], generating various free radicals such as OH·, H· from H_2_O, and C_1_· radicals [7–9] from CO_2_. Therefore, cavitation can induce various chemical reactions such as organic matter degradation, carbon dioxide reduction, and water splitting [4,6–9]. Theoretically, cavitation may have played a role in the generation of biogenic molecules [10–12].

Prebiotic synthesis represents a fundamental research frontier for elucidating origin of life. On primordial Earth, H_2_O functioned dualistically as both the universal solvent and a key reactant in biomolecule formation [13–15], while CO_2_ dominated the early atmosphere [16–18]. These primordial feedstocks—H_2_O and CO_2_—provided not only the essential reaction medium but also the chemical building blocks for critical biomolecules, with geochemical energy gradients driving their synthesis.

Ribose, a unique sugar in RNA, in the prebiotic realm is a critical stage for the subsequent molecular evolution in the chemical origins of life [19–21]. Understanding the prebiotic formation of ribose from H_2_O and CO_2_ in Earth’s primitive atmosphere is key to exploring the abiotic origin of biomolecules. Although the prebiotic formation [19–23] of ribose has been studied through the formose reaction, both experimentally [19–25] and theoretically [26,27], the direct formation of ribose from simple primordial molecules H_2_O and CO_2_ has not been explored.

**Figure 1.**
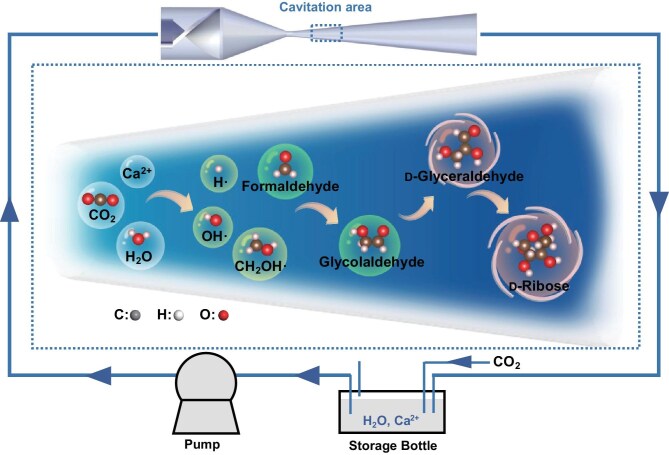
Schematic illustrations of ribose formation from H_2_O and CO_2_ by hydrodynamic cavitation. An aqueous reaction solution containing CaCO_3_ was pumped from a storage bottle into a Venturi tube, where it circulated within the reaction system while CO_2_ was bubbled into the solution.

Herein, we synthesized ribose from H_2_O and CO_2_  *via* hydrodynamic cavitation generated by a Venturi cavitator equipped with oblique blade (Fig. 1, see details in supplemental materials and Figs S1 and S2). Hydrodynamic cavitation can be replicated using water flow through a Venturi tube [28]. The ribose formation pathway was designed based on the principles of the formose reaction mechanism. Formaldehyde and free radical species such as H·, OH·, and CH_2_OH· radicals [8–11] were generated under the high temperature (1000–15 000 K) and pressure conditions (10–500 MPa) of hydrodynamic cavitation. CaCO_3_ reacted with H_2_O and CO_2_ to form Ca(HCO_3_)_2_, increasing dissolved CO_2_ concentrations and accelerating free radical formation [29]. Subsequent reactions led to the formation of glycolaldehyde, glyceraldehyde, and sugars, including ribose.

## RESULTS AND DISCUSSION

It is well established that the prebiotic ocean of H_2_O primarily consisted of NaCl at concentrations comparable to or exceeding those of modern oceans (0.5 M), along with ∼10 mM each of Ca^2+^, Mg^2+^, and K^+^ cations [30]. Temperature estimates for primitive Earth suggest a range of 30–80°C [16–18], while CO_2_ likely dominated the atmosphere [16,18,31]. Based on these parameters, sugar synthesis from H_2_O and CO_2_ was conducted in a Venturi tube under simulated prebiotic ocean conditions. Sugars were analyzed *via* gas chromatography-mass spectrometry after acetylation (Figs S3–S6). Yields were expressed as molar concentrations (μM) in the product solution.

**Figure 2.**
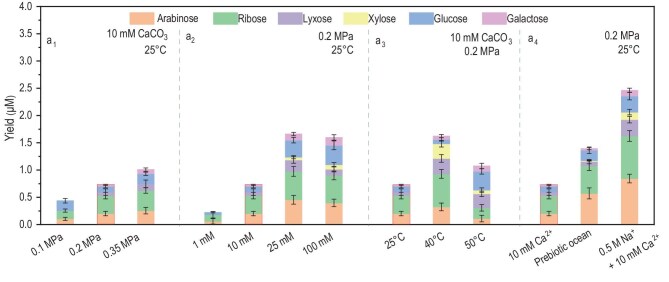
Synthesis of ribose by CO_2_ reduction in hydrodynamic cavitation. Yields of various sugars produced at different inlet pressures of 0.1, 0.2, and 0.35 MPa (a_1_), with different concentrations of CaCO_3_ at 1, 10, 25, and 100 mM (a_2_), at different temperatures of 25, 40, and 50°C (a_3_), and with 10 mM CaCO_3_, prebiotic ocean solution and 0.5 M NaHCO_3_ + 10 mM CaCO_3_ (a_4_). All reactions were carried out for 24 h, and the other reaction parameters are shown inset, respectively. The bars enclosed in the red boxes all represent the typical reaction conditions (0.2 MPa, 10 mM CaCO_3_, and 25°C). Error bars represent standard deviation in each experiment.

To investigate the effect of cavitation on the reaction, a series of experiments were performed at varying inlet pressures (Fig. 2a_1_). The total sugar yield increased with higher inlet pressures due to the enhanced cavitation energy, which facilitated greater CO_2_ conversion (Table S1). Total sugar yield of 1.01 μM was achieved with inlet pressure of 0.35 MPa, in which the yields of arabinose, ribose, lyxose, glucose, and galactose are 0.25, 0.37, 0.12, 0.19, and 0.08 μM, respectively. Control experiments using a straight tube instead of a Venturi tube resulted in negligible sugar production (Fig. S6), confirming that hydrodynamic cavitation was responsible for sugar synthesis.

Then, the reactions were also performed with varying concentrations of CaCO_3_ (Fig. 2a_2_ and Table S2). Increasing CaCO_3_ concentrations from 1 to 25 mM raised the yield of sugars from 0.22 to 1.65 μM, as CaCO_3_ enhanced CO_2_ dissolution and accelerated radical formation. Ribose yield increased from 0.12 to 0.52 μM. At 100 mM CaCO_3_, the yield plateaued, likely due to unconverted CaCO_3_ remaining in the solution. These results show that more concentration of dissolved Ca^2+^ cause higher yield of ribose from CO_2_ reduction by cavitation.

The effect of temperature on sugars and ribose yield was examined at 25, 40, and 50°C (Fig. 2a_3_ and Table S1). The total yield of sugars was higher at 40°C than that at 25°C, consistent with faster reaction rates at elevated temperatures. At 50°C, the ribose yield decreased while the hexose yield increased, likely due to the conversion of pentoses to hexoses at higher temperatures [25].

Simulations of prebiotic ocean conditions [28] (0.5 M NaCl, 10 mM CaCl_2_, 10 mM MgCl_2_, 10 mM KCl, and 10 mM NaHCO_3_) were conducted at 25°C and an inlet pressure of 0.2 MPa (Fig. 2a_4_). The sugar yields were higher than that of CaCO_3_ used solely as catalyst. The ribose yield was higher than that achieved using only CaCO_3_, as the weakly alkaline prebiotic ocean promoted ribose synthesis by generating additional OH· radicals during cavitation. The combination of CaCO_3_ with NaHCO_3_ resulted in ribose concentrations as high as 0.78 μM (Table S1). The absence of Cl⁻ in NaHCO_3_ solutions prevented sugar degradation *via* Cl^−^ attack, further enhancing ribose yield.

Intermediates and byproducts, including formaldehyde, methanol, ethanol, and formic acid, were characterized by ^1^H nuclear magnetic resonance (^1^H NMR) (Figs S7a and S8) and high-performance liquid chromatography (HPLC) (Table S2 and Fig. S9). Free radicals OH· and CH_2_OH· radicals [32] were detected *via* electron paramagnetic resonance (EPR) (Figs S7b, S10, and S11), which are the key intermediates for the formation of sugars by cavitation from CO_2_ and H_2_O. Glycolaldehyde and glyceraldehyde were observed using high-performance anion exchange chromatography with pulsed amperometry detection (HPAEC-PAD) (Figs S7c, S12, and S13). Peaks at 3.15 min corresponded to glycolaldehyde, while those at 6–11 min corresponded to C_5_–C_6_ sugars. As demonstrated in previous studies [19–27], glycolaldehyde and glyceraldehyde are essential intermediates in the formose reaction for sugar product formation.

To confirm the formation of ribose and glucose from CO_2_, isotope labeling experiments with ^13^CO_2_ in 10 mM Ca(OH)_2_ solutions were conducted. The ^13^C labeled products, PMP-derivatized for detection by HPLC-MS, confirmed that ribose originated from CO_2_ by hydrodynamic cavitation (Figs S14 and S15) [33]. The signal corresponding to ^13^C_5_-labeled-ribose and ^13^C_6_-labeled-glucose could be observed from HPLC-MS of isotope labeling experiments with ^13^CO_2_, which confirms the formation of ribose and glucose from CO_2_.

The products from hydrodynamic cavitation in a 0.5 M NaHCO_3_ + 10 mM CaCO_3_ solution were analyzed using ^1^H NMR. Methanol (CH_3_OH, *δ* = 3.35) and ethanol (CH_3_CH_2_OH, *δ* = 1.18 and 3.65) were detected (Figs S7a and S8), indicating that CH₂OH· radicals were generated not only from CO₂ reduction but also from CH_3_OH dehydrogenation in the presence of high Na⁺ concentration. The formation of CH₂OH· radicals from CH₃OH is thus plausible. Ethanol likely originated from the hydrogenation of glycolaldehyde or C_2_ byproducts by H· radicals. Formic acid was also detected, suggesting Cannizaro disproportionation [34] occurred due to the high Na⁺ concentration. Formaldehyde was observed under both reaction conditions (with and without Na⁺), but without Na⁺, only formaldehyde and trace amounts of methanol were detected (Table S2), indicating no Cannizaro disproportionation occurred.

EPR analysis of the reaction products in 0.5 M NaHCO_3_ and 0.5 M NaHCO_3_ + 10 mM CaCO_3_ solutions (Figs S7b, S10, and S11) revealed the presence of various free radicals, including OH· and CH₂OH· [32]. The signals of CH_2_OH· and OH· were lower in the presence of Ca^2+^, suggesting rapid consumption of CH_2_OH· in successive addition reactions catalyzed by Ca^2+^ (Fig. S7b). Only DMPO-CO_2_ and weak OH· signals were observed in pure water cavitation (Fig. S11). These results suggest that alkaline conditions promote OH· and CH₂OH· generation, supporting high sugar yields in prebiotic ocean and 0.5 M NaHCO_3_ + 10 mM CaCO_3_ systems. Glycolaldehyde and glyceraldehyde, known intermediates in the formose reaction, were detected by HPAEC-PAD (Figs S12 and S13) at an elution time of 3.15 min, while peaks around 6–11 min corresponded to C_5_–C_6_ sugars.

**Figure 3.**
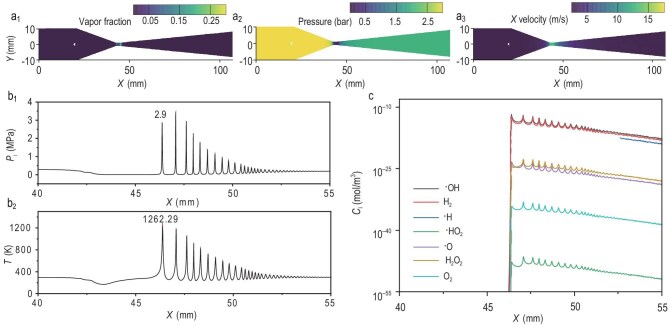
Numerical expression of hydrodynamic cavitation of pure H_2_O in the Venturi tube. (a) Vapor volume fraction (a_1_), absolute pressure (a_2_) and fluid velocity (a_3_). (b) Results of single bubble dynamic models showing variation in the Venturi tube with pure H_2_O, collapsing pressure (b_1_) and collapsing temperature (b_2_). (c) Production of free radicals.

To investigate the mechanism of ribose formation during hydrodynamic cavitation in a Venturi tube, temperature, pressure, phase state, and the resulting free radical species in the reaction solution were analyzed using a multiscale chemical model (MCM). The MCM integrates three key components: macroscopic computational fluid dynamics simulations, single-bubble dynamics, and chemical process simulations. These simulations were conducted for both pure water and seawater (Fig. 3, Tables S3 and S4, and Figs S16–S20).

The potential and intensity of cavitation are characterized by the cavitation number (*C_v_*). Cavitation is expected to occur when *C_v_* < 1 [35–37]. Simulations for pure water and seawater in the Venturi tube yielded *C_v_* values of 0.703 and 0.719, respectively, confirming the conditions necessary for cavitation. Figure 3a illustrates a contour map of vapor fractions for pure water (see also Fig. S16), showing a range from 0.05 to 0.27 within the *X* = 42.5–46.0 mm region, which corresponds to the occurrence of cavitation. The maximum vapor fraction of 0.27 was observed near *X* = 46.0 mm, suggesting that adiabatic bubble collapse at this location generates highly localized conditions of elevated pressure and temperature. These conditions within the cavitation bubbles were calculated to reach up to 2.9 MPa (Fig. 3b_1_) and 1262 K (Fig. 3b_2_) at *X* = 46.0 mm. Following this, the bubbles exhibited inertial oscillations accompanied by a gradual dissipation of energy between *X* = 46.0–55.0 mm.

As depicted in Fig. 3c, high temperatures and pressures around *X* = 46.0 mm led to the dissociation of H_2_O, generating free radicals such as OH·, O·, and H·. These radicals diffuse along vortex flows and participate in subsequent chemical reactions. Given that the salinity of the prebiotic ocean is comparable to modern seawater, hydrodynamic cavitation simulations for seawater were also conducted, yielding results similar to those observed for pure water (Figs S18–S20). In this process, the reaction of CO_2_ with OH· and H· produces CH_2_OH· and formaldehyde.

**Figure 4.**
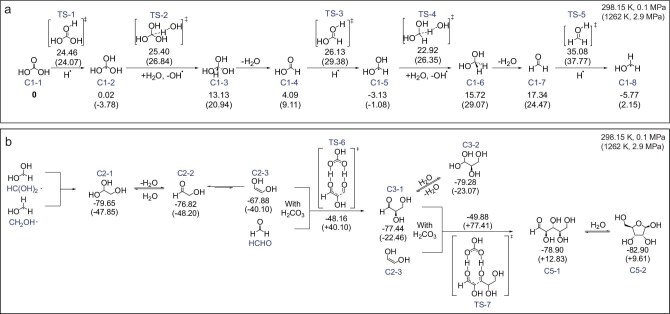
Density functional theory (DFT) calculation for the formation of ribose at 298.15 K and 0.1 MPa, and 1262 K and 2.9 MPa. (a) Pathway for the formation of aldehyde *via* various radicals. (b) Subsequent formose reactions for the formation of ribose.

Figure 4 illustrates the proposed pathway for ribose formation, supported by DFT calculations (Table S5). This pathway is based on the free radicals and molecules generated during the cavitation process [7,8]. The reaction mechanism is divided into two parts. The first involves radical reactions occurring under cavitation conditions (Fig. 4a), while the second comprises a series of condensation reactions catalyzed by H_2_CO_3_ (Fig. 4b). These reactions were modeled under two distinct conditions: room temperature and 0.1 MPa, and high-temperature (1262 K) and high-pressure (2.9 MPa) environment created by cavitation.

In hydrodynamic cavitation, H_2_O molecules dissociate into H· and OH· radicals under extreme temperature and pressure conditions. As shown in Fig. 4a, carbonic acid (C1-1) is formed from CO_2_ and H_2_O. Carbonic acid is reduced by H· *via* transition state TS-1, producing intermediate C1-2. Through a hydrogen atom transfer (HAT) process, orthoformic acid (C1-3) is generated *via* TS-2 and undergoes rapid dehydration to form formic acid (C1-4). Further reduction of C1-4 by H· produces intermediate C1-5 through TS-3. Methanediol (C1-6) is then formed *via* another HAT process (TS-4) and subsequently decomposed into formaldehyde (C1-7). Finally, reduction of C1-7 by H· *via* TS-5 generates CH_2_OH· (C1-8), a key intermediate. As a by-product, OH· radicals dimerize into H_2_O_2_, which decomposes into H_2_O and O_2_.

Figure 4b highlights the second phase of the reaction pathway, connecting radical reactions to sugar formation. The coupling of intermediates C1-5 and C1-8 results in the formation of hydrated glycolaldehyde (C2-1). Under aqueous conditions, C2-1 reversibly dehydrates into C2-2, which isomerizes into C2-3. An aldol reaction then occurs, where C2-3 condenses with formaldehyde (C1-7) under the catalytic action of H_2_CO_3_, producing glyceraldehyde (C3-1) *via* TS-6. A subsequent aldol reaction between C2-3 and C3-1, again catalyzed by H₂CO₃, leads to the formation of pentose (C5-1). This pentose molecule rapidly cyclizes into furanose (C5-2). Importantly, these condensation reactions are thermodynamically favorable at room temperature, suggesting that they occur under relatively mild conditions. This supports the hypothesis that ribose formation *via* cavitation is feasible and efficient, even in the absence of the extreme conditions typical of the radical formation phase. In the prebiotic world, nitrogen compounds such as NH_3_, HNO_2_, and HNO_3_ were also present [38,39]. These nitrogenous species could serve as the nitrogen source for the formation of amino acids and nucleotides, thereby facilitating the formation of ribose as well.

## CONCLUSION

In this study, we present a phenomenon for the formation of ribose directly from H_2_O and CO_2_  *via* hydrodynamic cavitation, under conditions closely resembling those of the prebiotic ocean of H_2_O. According to MCM calculations, the entire reaction is driven by the extremely high energy released during the collapse of hydrodynamic cavitation bubbles. This intense energy initiates the formation of H·, OH·, and C_1_· radicals from H_2_O and CO_2_, which subsequently lead to the production of formaldehyde and CH_2_OH· intermediates, followed by subsequent formation of glycolaldehyde and glyceraldehyde for formation of ribose. These discoveries demonstrate hydrodynamic cavitation as a fundamental mechanism for prebiotic formation of ribose. Moreover, flowing water has been discovered to be able to drive biomolecular formation by itself in this work.

## Supplementary Material

nwag182_Supplemental_File

## Data Availability

The data supporting this study are available within the paper and the Supplementary Information. All other relevant source data are available from the corresponding author upon reasonable request.
