# Supplementary material for: Hydrodynamic cavitation for prebiotic ribose formation from H2O and CO2
Source: Natl Sci Rev. 2026 Mar 23;13(11):nwag182. doi: 10.1093/nsr/nwag182 (PMC13281097; doi:10.1093/nsr/nwag182)
Supplement: nwag182_Supplemental_File [file nwag182_supplemental_file.pdf]

Supplementary Materials for

Hydrodynamic Cavitation for Prebiotic Ribose Formation from H_2O and CO_2

Yuxi Fang[1]*, Yicong Luo[1], Wei Ding[2], Diwen Ying[2], Wanning Zhang[1], Jiaxin Song[1], Wanbin Zhang[1]* and Jinping Jia[1, 2]*

*Corresponding author. Email: sjtu15901600323@sjtu.edu.cn; wanbin@sjtu.edu.cn; jpjia@sjtu.edu.cn

The PDF file includes:

Materials
Supplementary Text
Figures S1 to S20
Tables S1 to S5

Table of Contents

Materials S1

Venturi cavitator setup S2

Characterization of products S3

Numerical studies S12

DFT calculations S19

Materials

Sodium bicarbonate (99.5 %), sodium chloride (99.99 %), magnesium chloride (99%), pottasium chloride (99.9 %), D-ribose (99 %), L-ribose (99 %), D-arabinose (98 %), L-arabinose (98 %), D-xylose (98 %), L-xylose (98 %), D-lyxose (99 %), L-lyxose (99 %), D-glucose (99 %), L-glucose (99 %), D-galactose (98 %), L-galactose (99 %), 5,5-Dimethyl-1-pyrroline-N-oxide (DMPO, 98%), acetone (99.9 %), pyridine (99.5 %), acetic anhydride (99 %), hydroxylamine hydrochloride (99 %) and chloroform (99.0 %) were purchased from Adamas Ltd. Calcium carbonate (99.999 %) and calcium chloride (99.99 %, metal basis) was purchased from Shanghai Aladdin Biochemical Technology Co., Ltd.

Methods

Venturi cavitator setup

Synthesis runs were performed in an experimental setup as shown in Fig. S1. It consists of a reaction bottle (500 mL) and jet flow loop. The solution temperature was maintained at 25~50 °C by a coil pipe in a thermostatic bath and an aeration friox was equipped to bubble CO_2 (99.999%) into the reaction solution. Aqueous solution in the reactor was circulated through the jet flow loop, which was driven by a plunger pump (2.2 kW) with a 304 stainless steel pump head. The pump outlet was connected to the Venturi tube through lines, throttle valves and pressure gauge at the lines were provided for adjusting and measuring the pressure in the main line. A Venturi tube as shown in Fig. S2 was made of polymethyl methacrylate and equipped with oblique blade. The inner diameter of throat of the Venturi tube is 2 mm. Its expansion and contraction angles are both 20°.

Synthesis of ribose by hydrodynamic cavitation

Typical experiments were performed for 24 h with 1 L of reaction solution (300 mL in reaction bottle + 700 mL in pipes and pump) at 25 °C. In the typical synthesis, 10 mmol $CaCO_3$ was mixed with 300 mL Millipore water in reaction bottle. After bubble CO_2 into the reaction solution for 10 min, solution was looped for 24 h with continuous CO_2 flow, with inlet pressure of 0.2 MPa and flow rate of 3.2 LPM (lines per minute) of fluid. 300 mL of reaction solution was collected and applied to a column filled with ~5 mL of cation exchange resin in hydrogen form (Amberlite IRC120, Sigma-Aldrich). The column was eluted with the reaction solution after eluted with water. Finally, the eluate was lyophilized to remove water for next characterization.

For NMR characterization, the scale-up reaction was performed for 72 h with 2.7 L of reaction solution (2 L in reaction bottle + 700 mL in pipes and pump) at 25 °C.

For proving the mechanism of condensation reaction to sugars from formaldehyde and glycolaldehyde. The experiment was performed in 1L 100 mM formaldehyde and 0.1 mM glycolaldehyde (300 mL in reaction bottle + 700 mL in pipes and pump) at 25 °C. After bubble CO_2 into the reaction solution for 10 min, solution was looped for

24 h with continuous CO_2 flow, with inlet pressure of 0.2 MPa and flow rate of 3.2 LPM of fluid.

Analysis of various sugars

Arabinose, ribose, xylose, lyxose, glucose and galactose were detected by GC-MS (7890B-7000D, Agilent) equipped with a column (Rtx-5MS, Restek 30 m × 0.25 mm × 0.25 mm) after derivatized [1]. 10 mg of sample and 12 mg of hydroxylamine hydrochloride was dissolved in 0.6 mL of pyridine in vial. Then vial was sealed and heated at 90 °C for 30 min. After cooled to room temperature, 0.6 mL of acetic anhydride was added. Then vial was sealed and heated at 90 °C for 30 min. After cooled to room temperature, acetic acid and pyridine were removed by nitrogen purging for 4 h at 50 °C water bath. The sample was dissolved in 2 mL of chloroform. The detection of samples was conducted in EI mode with a mass range of 35-550. Then, a 2 μL sample was injected and flow with a flow rate of 1.0 mL/min and helium as a carrier gas. Inlet temperature is 280 °C and initial oven temperature is 130 °C. Oven temperature is finally heated to 280 °C with rate of 3 °C /min.

Isotope labeling

The isotope labelling experiment was performed in same hydrodynamic cavitator in synthesis of ribose by hydrodynamic cavitation. Volume of $^{13}CO_2$ (2 MPa, gas with 99.967% ^{13}C in 0.5 L gas cylinder) was not enough for performing the isotope experiment for 24 h. Therefore, in ^{13}C labelling synthesis, 10 mmol $Ca(OH)_2$ was mixed with 300 mL Millipore water in the reaction bottle. After bubbled $^{13}CO_2$ into the reaction solution for 5 min, solution was looped for 6 h with continuous $^{13}CO_2$ flow, with inlet pressure of 0.2 MPa and flow rate of 3.2 LPM of fluid. 300 mL of reaction solution was collected and applied to a column filled with ~5 mL of cation exchange resin in hydrogen form (Amberlite IRC120, Sigma-Aldrich). The column was eluted with the reaction solution after eluted with water. Finally, the eluate was lyophilized to remove water for next characterization.

The ^{13}C labelled sample was analyzed by HPLC-MS (Acquity UPLC & XEVO G2-XS QTOF, Waters, United States) to confirm the formation of ^{13}C labelled sugars. The experimental samples (if standard ribose and glucose were measured, 1 mg of each was used) were dissolved in 0.1 mL of water and then mixed with 0.1 mL of 0.5 M PMP methanol solution. The mixture was heated at 70 °C for 30 min. Subsequently, 1 mL 10% acetic acid aqueous solution and 1mL of chloroform were added. After that, the sample was extracted with a separatory funnel (aqueous phase retained), washed with 1 mL chloroform by shaking, re-extracted, centrifuged (8,000 rpm, 10 min), and the aqueous phase was collected via syringe and filtered through a 0.22 μm membrane to obtain the final test sample. Chromatographic experiments used an Agilent HPLC coupled to triple-quadrupole mass detector with an electrospray ionization (ESI) source (Agilent 6495, Agilent Technologies, Santa Clara, CA, USA). BEH C18 column (2.1 mm × 150 mm, 1.7 μm) was used for chromatographic isolation. The mobile phase was 0.1% formic acid (FA) acetonitrile (ACN) solution (A) and 0.1% FA-H_2O solution(B) at a flow rate of 0.3 mL/min. Separation used gradient elution from 1% A to 100% A. The column temperature was set at 45 °C, and the injection volume was 1 μL. The MS was operated with ESI in positive ionization mode. MS analysis was detected in scan mode with a mass range of 50-600. The settings of MS included a nebulizer pressure of 50 psi. The drying gas flow rate was 10 L/min, and the temperature was 350 °C. The value of the fragmentor was 135 V with capillary voltage at 4000 V in positive ion scan mode.

Numerical procedure

In this study, MCM simulation was conducted, which consists of three components: CFD, single bubble dynamics and chemical process simulation.

Multiphase steady-state 3D CFD simulations were conducted using ANSYS Fluent (version 2020 R1). Schnerr-Sauer [2] was chosen as the cavitation model to obtain the bulk flow characteristics like bulk pressure, bulk velocity and vapor volume fraction throughout the domain. The turbulence model was described by the realizable k-ε turbulence [3] with standard wall treatment. The boundary conditions were defined as

pressure inlet and a pressure outlet, respectively. The gauge pressure at inlet was set at 0.2 MPa whereas the outlet gauge pressure value was specified at 0.1 MPa corresponding to the experimental conditions. Pressure-velocity coupling was carried out by PISO algorithm and the PRESTO! scheme was used for pressure discretization. The second-order upwind discretization utilized for both the turbulence kinetic energy and turbulence dissipation rate for improved accuracy. The simulation is considered to have achieved convergence when all residuals fall below $1e^{-5}$.

Discrete phase model (DPM) is used in this study to understand the cavitation nucleus behavior within the flow domain. The injected inert particles are massless and discrete random walk model is also utilized in calculation considering the effect of turbulence fluctuations on the cavitation nucleus. Once the simulation is converged, the data of pressure vs flow time is exported to solve single bubble dynamics model [4-7].

The physical and chemical properties of pure water and seawater are slightly different. We set the saturated steam fraction of seawater as 3166 Pa in fluent, and the density and viscosity of seawater under 25 °C are 1023.288 kg/m^3 and 0.00102 kg/m·s, respectively.

Python 3.10 along with Scipy 1.11.1 was employed as the computational engine to solve the ordinary differential equations using the ode45 method for single bubble dynamics and chemical process simulation. The initial radius of the cavitation bubble was set at 100 μm, with an initial bubble wall velocity of 0 and an initial temperature of 298.15 K. The initial partial pressure of the water molecules is the saturated vapor pressure corresponding to the initial temperature, with the initial concentrations of all chemical components set to zero.

For freshwater and seawater, the volumetric flowrate is 7.44×10^{-5} m^3/s and 7.335×10^{-5} m^3/s, respectively. Based on the geometry of the Venturi throat, the corresponding linear flow rates were calculated to be 23.7 m/s and 23.4 m/s. Inlet pressure and outlet pressure was 0.2 MPa and 0.1 MPa, respectively. The cavitation number was calculated as:

$$\sigma_{fresh} = \frac{P_{out} - P_v}{\frac{1}{2}\rho v^2} = \frac{201325 - 3540}{\frac{1}{2} * 1000 * 23.7^2} = 0.703$$

$$\sigma_{sea} = \frac{P_{out} - P_v}{\frac{1}{2}\rho v^2} = \frac{201325 - 3540}{\frac{1}{2} * 1000 * 23.4^2} = 0.719$$

DFT calculation procedure

Computations were performed using the Gaussian 09 (revision D.01) suite of quantum chemical program. All structures are optimized in an implicit solvent model using the M062X [8] hybrid functional with usage of empirical Grimme's dispersion correction [9] (GD3-BJ). In optimization, all atoms are described with def2-SVP [10]. All structures are in a local minimum potential energy surface with zero imaginary frequency or at the first order saddle point (transition state) on the potential surface with one imaginary frequency. A global multiplicative harmonic frequency scaling factor for M062X/def2-SVP basis set, 0.9762 [11], were used as a correction for calculated harmonic frequencies and thermal data. The contribution of low frequency vibration is modified by quasi-harmonic approximation proposed by Grimme [12] considering the contribution of low frequency vibration (<100.0 cm^{-1}) to the partition function, using the free-rotor approximation. For those above this threshold, the RRHO approximation is retained. All frequency and thermal corrections are computed using Shermo [13]. The single point energies were calculated at revDSD-PBEP86-D3(BJ)/cc-pVTZ level of theory [14]. The effect of a solvent continuum, in THF, was evaluated using the Cramer−Truhlar continuum solvation model that describes the electrostatic interaction and nonpolar interaction between solvent and solute, named as SMD [15]. The calculation of hydrogen radical and hydroxy radical was carried out with the mixture of implicit and explicit model.

Characterizations

HPAEC-PAD was performed by Dionex ICS-5000. The samples were run on a CarboPac PA20 IC column using a 0.4 mL/min isocratic gradient as follows: with 10

mM NaOH for 30 min, 100 mM NaOH for 5 min, and 10 mM NaOH for 5 min. A gold electrode in carbohydrate quad potential mode was employed as the detector. Upon injection, the elution is carried with 10 mM NaOH for 30 min, 100 mM NaOH for 5 min, and 10 mM NaOH for 5 min. Runs were compared with standards of (2 to 6 carbons) biologically relevant sugars: arabinose, glucose, ribose, fructose, acetaldehyde, and formaldehyde were employed to identify peaks in the trace.

^{1}H NMR spectroscopy was performed by Bruker Advance-III 500. Production yield was estimated from the report of 1H NMR using DMSO. 0.01408 μmol DMSO was dissolved in 600 μL measured liquid (50 μL DMSO solution + 100 μL D_2O + 450 μL reaction solution) as an internal standard substance.

For the detection of formaldehyde, HPLC was performed by Thermo Fischer Scientific, U3000. The 10 μL of samples were run on a Hypersil GOLD C18 column (150 mm × 4.6 mm, 5 μm) with flow rate of 1 mL/min, UV detector for 350 nm and 25 °C of column temperature. Mobile phase is deionized water/acetonitrile = 40/60. Before the measurement, formaldehyde was derivatized as follows: 1.25 mL of reaction solution was mixed with 2 mL acetonitrile. Then, the mixture was added with 0.25 mL 2,4-dinitrophenylhydrazine + phosphoric acid solution (51 mg 2,4-dinitrophenylhydrazine + 10 mL concentrated phosphoric acid). The mixture was reacted at room temperature for 2 h.

For the detection of formic acid, ethylene glycol, acetic acid and dihydroxyacetone. HPLC was performed by Waters, e2695. The 10 μL samples were run on a BIO-RAD-Aminex HPX-87H column (300 mm × 7.8 mm) with flow rate of 0.5 mL/min, RID detector and 50 °C of column temperature. Mobile phase is 5 mM H_2SO_4.

The EPR spectrometer (Bruker EMXplus-9.5/12) settings were the following: microwave frequency 9.759 GHz, modulation amplitude 1.00 G, magnetic field scan 154.7 G, sweep time 41.94 s, time constant 2.56 ms, and five accumulations. 1~2 mL of reaction solution was directly ejected from throttle valve connected to outlet of Venturi tube, then immediately mixed with 20 μL DMPO.

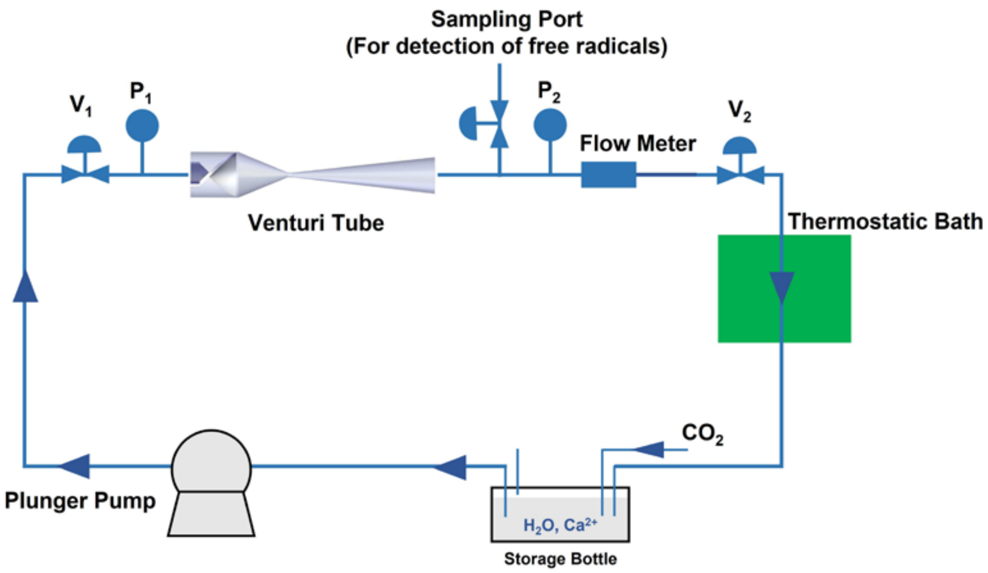

Fig. S1. Schematic sketch of Venturi cavitator.

The hydrodynamic cavitation system consists of a plunger pump, a Venturi cavitator, a thermostatic bath, and a storage bottle. The pump (Model: BM910-C28, Nantong Desto) provides a flow rate of 19 L/min with a power of 2.2 kW and is constructed from 316L stainless steel. The thermostatic bath (Model: MDC-0510, Dr Ant) maintains a constant temperature, utilizing a circulation pump with a flow rate of 10 L/min and a power of 1.2 kW. All connectors and tubing within the system are also made of 316L stainless steel. The core component is the Venturi cavitator consists of a Venturi tube equipped with oblique blade, plunger pump, storage bottle and thermostatic bath. The Venturi tube comprises a gradually narrowing inlet section, a constricted throat segment and a gradually expanding outlet section (see Fig. S2 for detail dimensions).

Cavitation could occur when high-speed flows pass through throat of the Venturi tube due to the pressure drop in the constriction of throat. The high temperature and high pressure are generated as the cavitation bubbles collapse adiabatically under the influence of the recovered hydraulic pressure. The extent of cavitation can be controlled by adjusting the inlet pressure of reaction fluids.

Aqueous reaction fluid containing Ca^{2+} was pumped from storage bottle into the Venturi tube and looped in the reaction system, with CO_2 being continuously bubbled into the solution. The extent of cavitation was controlled by adjusting inlet pressure (P_1, 0.1~0.6 MPa) at the inlet of the Venturi tube. The inlet pressure was regulated by pumping pressure of a plunger pump. The outlet pressure (P_2) was also measured to calculate the cavitation number (Cv). The flow rate was measured by flow meter, which is increases with increasing inlet pressure. The temperature (0~60 °C) was controlled by a thermostatic bath. A coil pipe was installed in thermostatic bath to maintain a stable temperature. The sample for detection of free radicals was taken at sampling port connected to the outlet of the Venturi tube. The samples for determining the yield of sugars and other intermediates were collected by collecting the entire reaction solution from storage bottle.

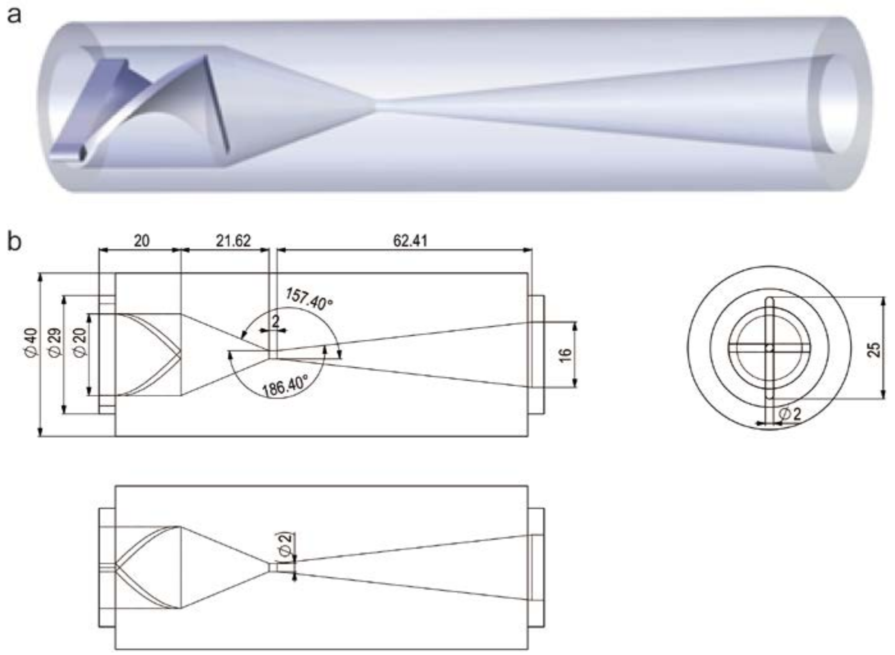

Fig. S2. (a) 3D Schematic illustrations of a Venturi tube. (b) Three views of a Venturi tube with detailed size. The unit of size is millimeters.

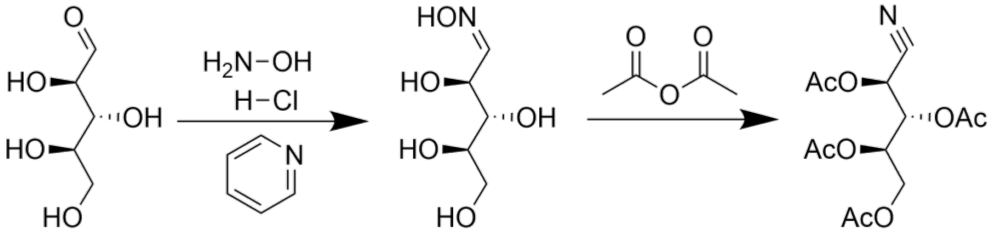

Fig. S3. Scheme for acetylation of carbohydrates (e.g., ribose is depicted here) to perform quantification of sugars by GC-MS.

The aldononitrile acetate derivatization is widely used for gas chromatography (GC) and GC–mass spectrometry (GC–MS) analyses of carbohydrates. The derivatization procedure gives a unique peak for aldose, such as ribose. Initially, sugar oxime can be obtained after hydroxylamine hydrochloride treatments of aldose. Subsequently, the aldononitrile and acetylation steps were carried out for the sugars by adding acetic anhydride.

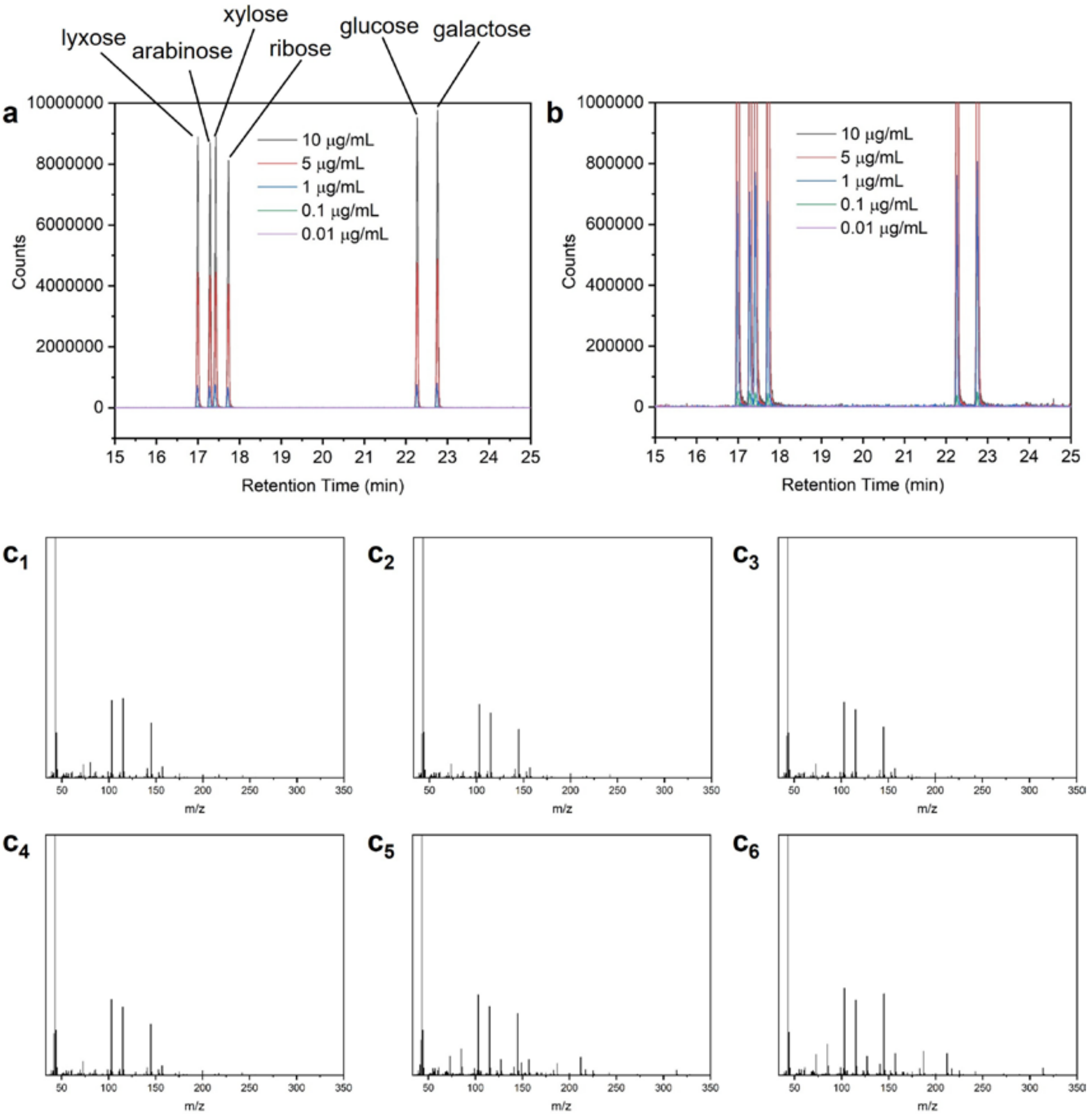

Fig. S4. GC-MS (extracted ion, m/z = 145.0) of standard samples with different concentrations for quantification of sugars (a) and its zoomed spectra (b). Corresponding MS peaks of lyxose with retention time = 17.00 min (c_1), arabinose with retention time = 17.30 min (c_2), xylose with retention time = 17.42 min (c_3), ribose with retention time = 17.74 min (c_4), glucose with retention time = 22.27 min (c_5) and galactose with retention time = 22.77 min (c_6).

A series of acetylated standard samples of lyxose, arabinose, xylose, ribose, glucose and galactose were analyzed by GC-MS. Each peak corresponding to the acetylated sugars was well-resolved, enabling accurate quantification of these sugars.

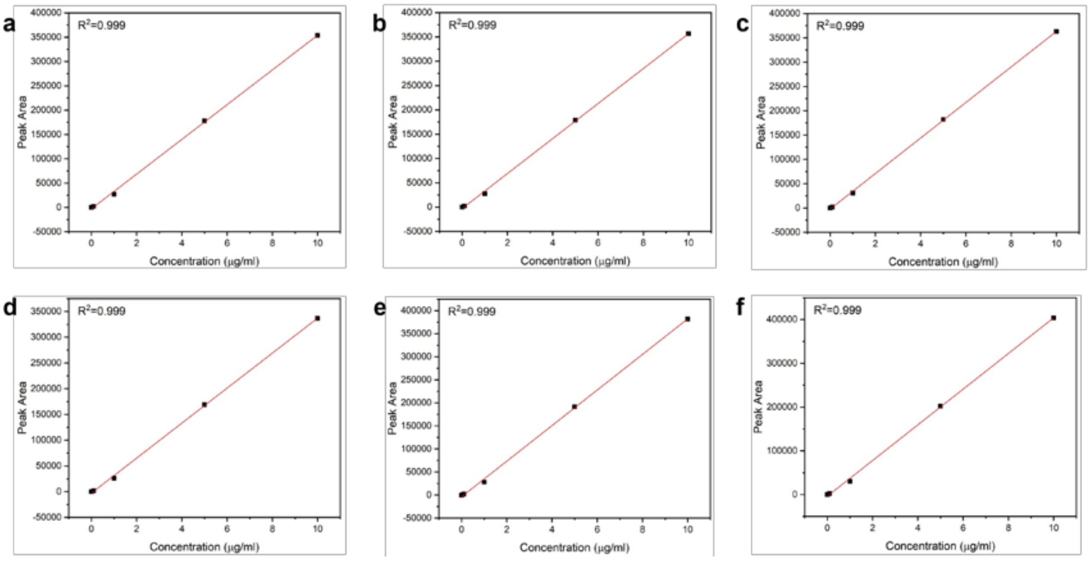

Fig. S5. Standard curves for lyxose (a), arabinose (b), xylose (c), ribose (d), glucose (e) and galactose (f).

Standard curves in Fig. S5 were generated based on GC-MS analyses of a series of standard solution containing lyxose, arabinose, xylose, ribose, glucose and galactose.

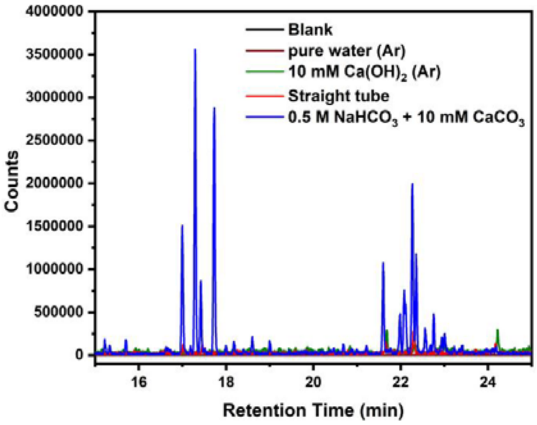

Fig. S6. GC-MS analysis (extracted ion, m/z = 145.0) of samples from control experiments, blank (black line, with no injection), with pure water bubbling with Ar (brown, control), 10 mM $Ca(OH)_2$ bubbling with Ar (green, control), 0.5 M $NaHCO_3$+10 mM $CaCO_3$ solution using straight tube instead of Venturi tube (red, control) and 0.5 M $NaHCO_3$+10 mM $CaCO_3$ solution (blue, reaction sample).

A series of control experiments were conducted to confirm that sugars were indeed converted from CO_2. No products were generated from the reaction in pure water, which indicate Ca^{2+} is the necessary for catalytic formation of ribose from CO_2. Also, no products was generated from the reaction in 10 mM $Ca(OH)_2$ under continuous Ar flow, indicating that there were no contribution of contaminations in water and environment on formation of ribose. Additionally, no sugar products were detected from the reaction using the straight tube instead of the Venturi tube. These results

demonstrate that cavitation is the primary driving force for formation of ribose in the reaction system.

Table S1. Yields of sugars in the series of samples shown in Fig. 2.

Samples	Arabinose (μM)	Ribose (μM)	Lyxose (μM)	Xylose (μM)	Glucose (μM)	Galactose (μM)
0.1 MPa[a]	0.10	0.15	n/a	n/a	0.18	n/a
0.2 MPa[a]	0.20	0.33	0.06	n/a	0.11	0.02
0.35 MPa[a]	0.25	0.37	0.12	n/a	0.19	0.08
1 mM[a]	0.06	0.12	n/a	n/a	0.04	n/a
25 mM[a]	0.45	0.52	0.21	0.05	0.31	0.11
100 mM[a]	0.40	0.51	0.11	0.08	0.36	0.15
40 °C[b]	0.32	0.61	0.28	0.27	0.08	0.06
50 °C[b]	0.11	0.20	0.26	0.07	0.34	0.10
prebiotic ocean[c]	0.57	0.51	0.07	0.017	0.191	0.031
0.5 M $NaHCO_3$ + 10 mM $CaCO_3$[c]	0.84	0.78	0.30	0.14	0.30	0.10

[a]Experiments with 10 mM $CaCO_3$ solution under 25 °C for 24 h.

[b]Experiments with 10 mM $CaCO_3$ and inlet pressure of 0.2 MPa for 24 h.

[c]Experiments with inlet pressure of 0.2 MPa under 25 °C for 24 h.

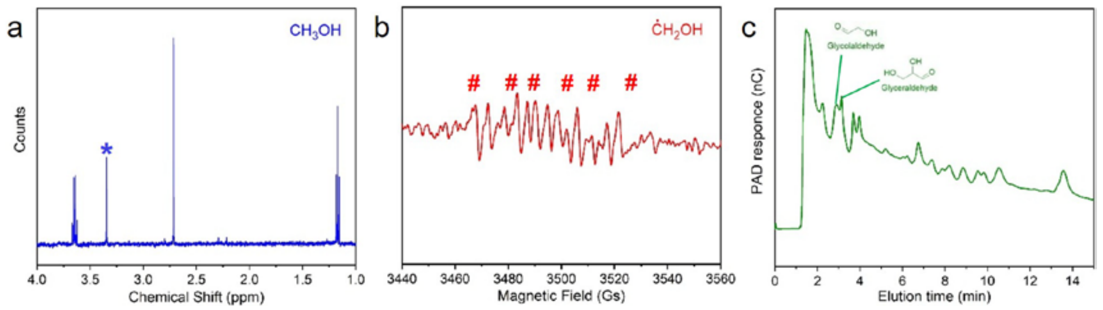

Fig. S7. Characterizations for possible intermediates and free radicals in the pathway of sugar formation of sugars by hydrodynamic cavitation. (**a**) **^{1}H** NMR of products in 0.5 M $NaHCO_3$ + 10 mM $CaCO_3$ solution. (**b**) EPR of free radicals in 0.5 M $NaHCO_3$ solution, added with DMPO as free radical scavenger. (**c**) HPAEC PED of products in 0.5 M $NaHCO_3$ + 10 mM $CaCO_3$ solution.

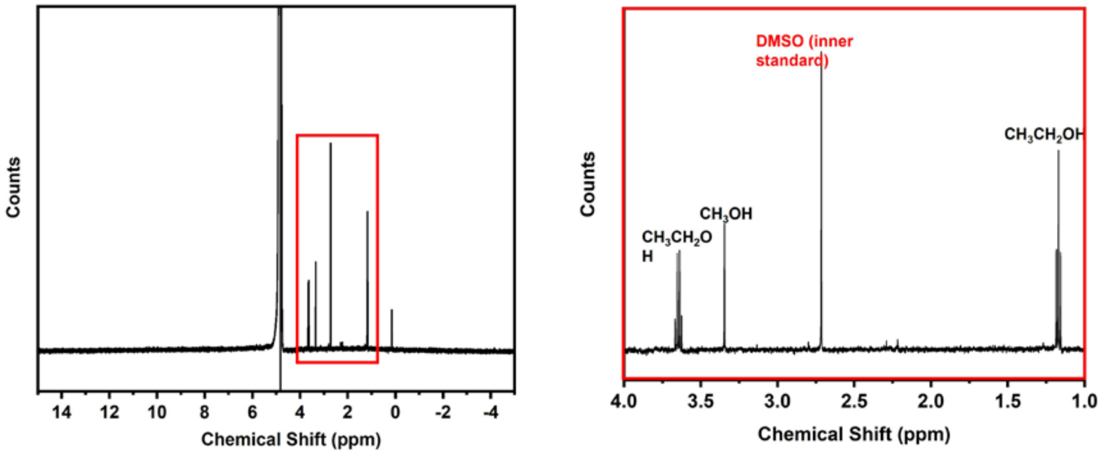

Fig. S8. ^{1}H NMR of the byproducts from the reaction in 0.5 M $NaHCO_3$+10 mM $CaCO_3$.

18.8 μM of methanol and 65.3 μM of ethanol were the main byproducts, indicating the large energy occurred by cavitation also cause the continuous reduction of CO_2 to alcohols. As shown in Fig. 3c, the H· radicals would be easily generated by hydrodynamic cavitation. These H· radicals were speculated to react with part of intermediates by hydrodynamic cavitation to form methanol and ethanol. On the other hand, methanol is also the reactant in the process of formation of sugars while ethanol could not be consumed in this reaction. Thus, the amount of produced ethanol was greater than that of methanol.

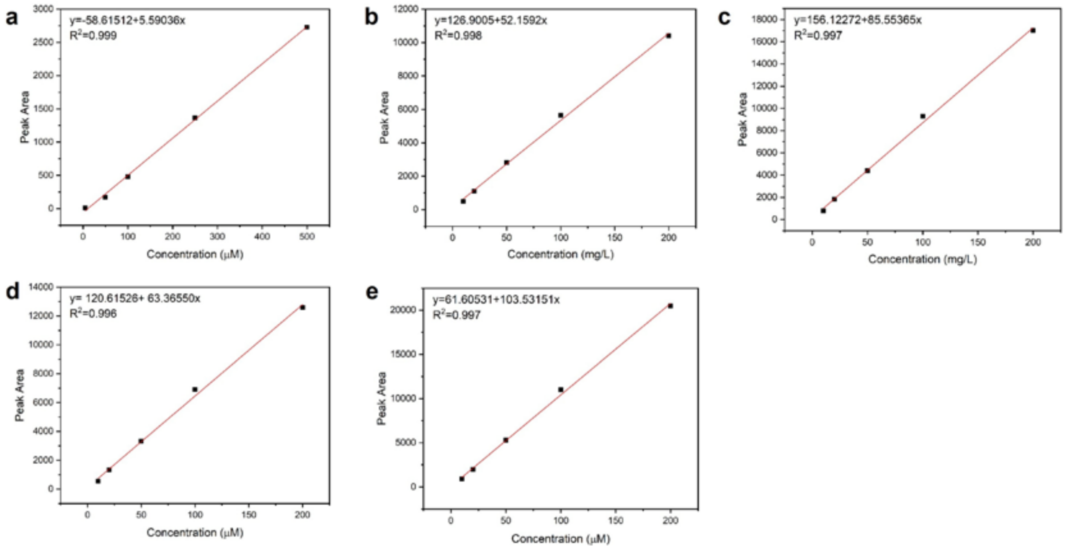

Fig. S9. Standard curves for HPLC of formaldehyde (a), formic acid (b), ethylene glycol (c), acetic acid (d) and dihydroxyacetone (e).

Standard curves in Fig. S9 were generated based on HPLC analyses of a series of standard solutions containing formaldehyde, formic acid, ethylene glycol, acetic acid and dihydroxyacetone.

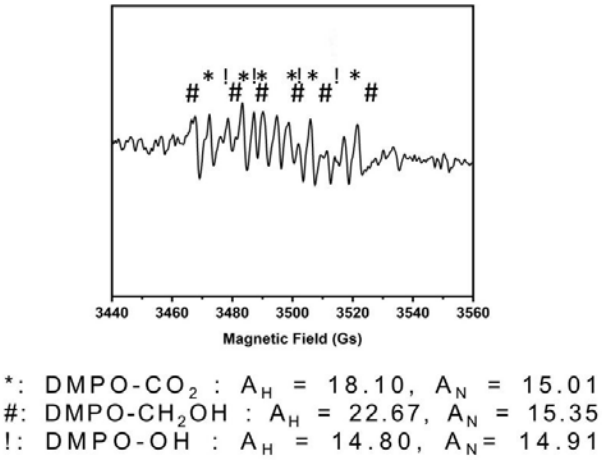

Fig. S10. EPR of free radicals from hydrodynamic cavitation in 0.5 M $NaHCO_3$ under CO_2 atmosphere, added with DMPO as free radical scavenger with signals of DMPO-CO_2, DMPO-CH_2OH and DMPO-OH marked.

As shown in Fig. S10, numerous peaks corresponding to free radicals scavenged by DMPO could be observed in EPR spectrum. The main peaks could be attributed to DMPO-CO_2, DMPO-CH_2OH and DMPO-OH. Although the EPR spectrum information is somewhat obscured by complexity of composition of free radicals in 0.5 M $NaHCO_3$, the presence of minor peaks corresponding to DMPO-O_2 and DMPO-OCH_2CH_3.

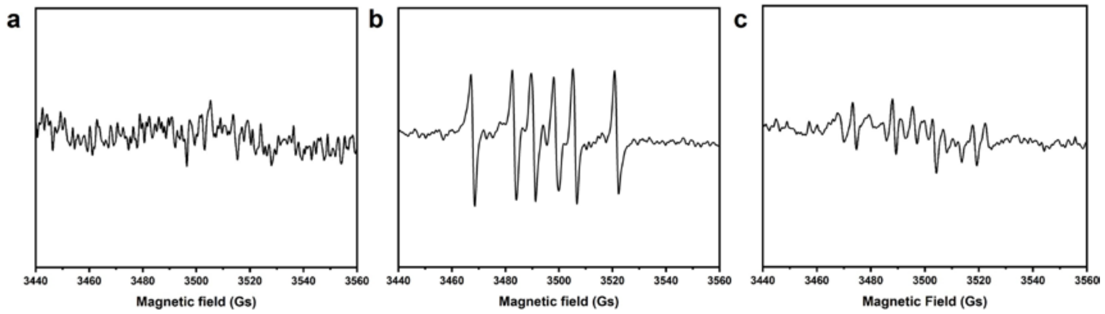

Fig. S11. EPR of free radicals from hydrodynamic cavitation in 0.5 M $NaHCO_3$+10 mM $CaCO_3$ (a) and pure water (b) under CO_2 atmosphere, in 100 mM CH_3OH (c), added with DMPO as free radical scavenger.

As shown in Fig. S11a, the presence of Ca^{2+} in 0.5 M $NaHCO_3$ significantly reduces the number of free radicals detected by EPR compared to that of 0.5 M $NaHCO_3$. It may be attributed to Ca^{2+} catalyzing the formation of sugars by consuming OH· and CH_2OH· radicals, thereby decreasing the number of free radicals available for scavenged by DMPO. Additionally, in pure water, only the abundant DMPO-CO_2 and a few DMPO-OH peaks were observed, indicating that the presence of Na^+ or weak alkaline condition of reaction solution promotes the formation of free radicals OH· and CH_2OH· by increasing concentration of OH^- ions. As shown in Fig. S11c, signal of DMPO-CH_2OH· could be observed in the EPR, which indicates CH_2OH· radical also could be generated from MeOH dehydration.

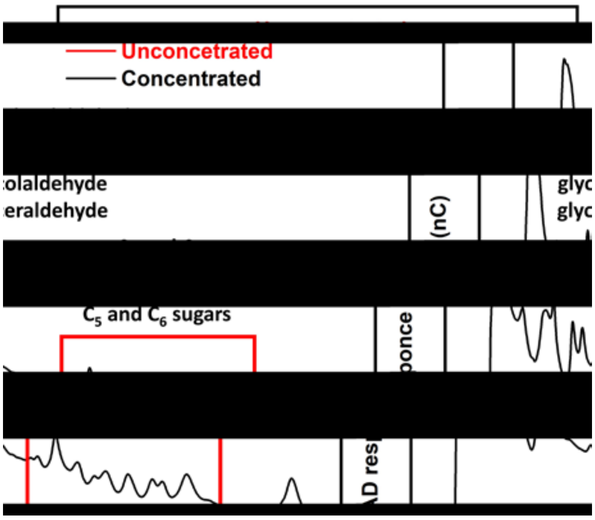

Fig. S12. Detailed HPAEC PED of products from hydrodynamic cavitation in 0.5 M $NaHCO_3$ + 10 mM $CaCO_3$ solution.

Glycolaldehyde and glyceraldehyde were observed from HPAEC PED in both unconcentrated and concentrated solution. The peaks attributed to C5 and C6 sugars were also observed in the range of 6-11 min.

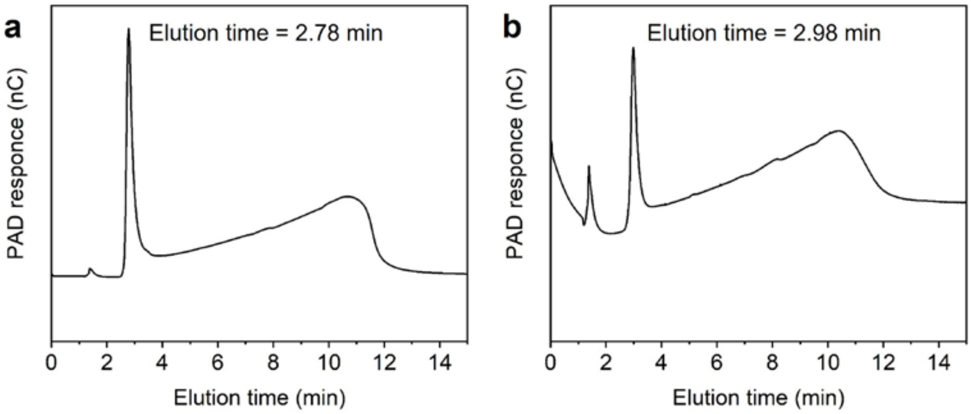

Fig. S13. HPAEC-PED of 500 μg/ml of glycolaldehyde (a) and glyceraldehyde (b) aqueous solution.

Table S2. Yields of byproducts in the series of samples shown in Fig. 2.

Samples	Methanol (μM)	Ethanol (μM)	Formaldehyde (μM)	Formic acid (μM)	Ethylene glycol (μM)	Acetic acid (μM)	Dihydroxyacetone (μM)
0.1 MPa[a]	n/a	n/a	11.2	n/a	n/a	n/a	n/a
0.2 MPa[a]	1.2	n/a	13.4	n/a	n/a	n/a	n/a
0.35 MPa[a]	2.6	n/a	15.8	n/a	n/a	n/a	n/a
25 mM[a]	1.1	n/a	17.5	n/a	n/a	n/a	n/a
40 °C[b]	4.1	n/a	15.3	n/a	n/a	n/a	n/a
0.5 M $NaHCO_3$ + 10 mM $CaCO_3$[c]	18.8	65.3	17.2	20.1	n/a	n/a	n/a

[a]Experiments with 10 mM $CaCO_3$ solution under 25 °C for 24 h.

[b]Experiments with 10 mM $CaCO_3$ and inlet pressure of 0.2 MPa for 24 h.

[c]Experiments with inlet pressure of 0.2 MPa under 25 °C for 24 h.

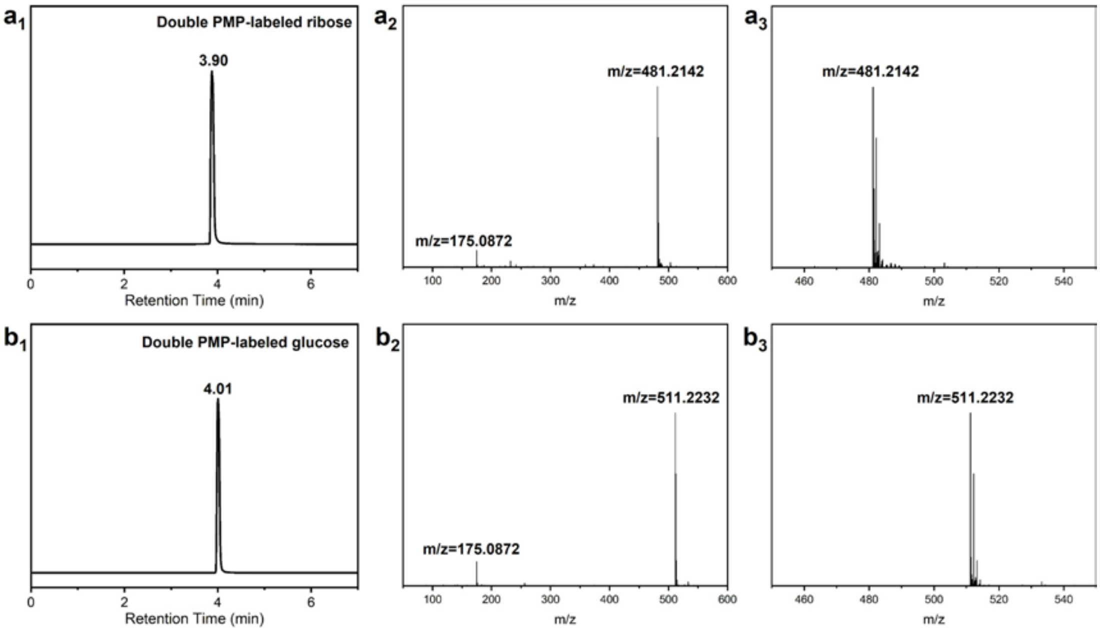

Fig. S14. HPLC-MS of standard double PMP-labeled ribose (extracted ion =481.21 (m/z)) (a_1) with full mass spectrum (a_2) and zoomed mass spectrum (a_3), and standard double PMP-labeled glucose (extracted ion =511.22 (m/z)) (b_1) with full mass spectrum (b_2) and zoomed mass spectrum (b_3).

As shown in Fig. S14, double PMP-labeled ribose exhibits a retention time of 3.90 min in the HPLC chromatogram (Fig. S14a_1), with a corresponding peak at m/z= 481.2142 (Fig. S14a_2 and a_3). Similarly, double PMP-labeled glucose shows a

retention time of 4.01 min (Fig. S14b_1) and a peak at m/z= 481.2142 (Fig. S14b_2 and b_3). Notably, the peak at m/z= 175.0872 observed in both spectra corresponds to the PMP fragment, a characteristic moiety of both derivatives.

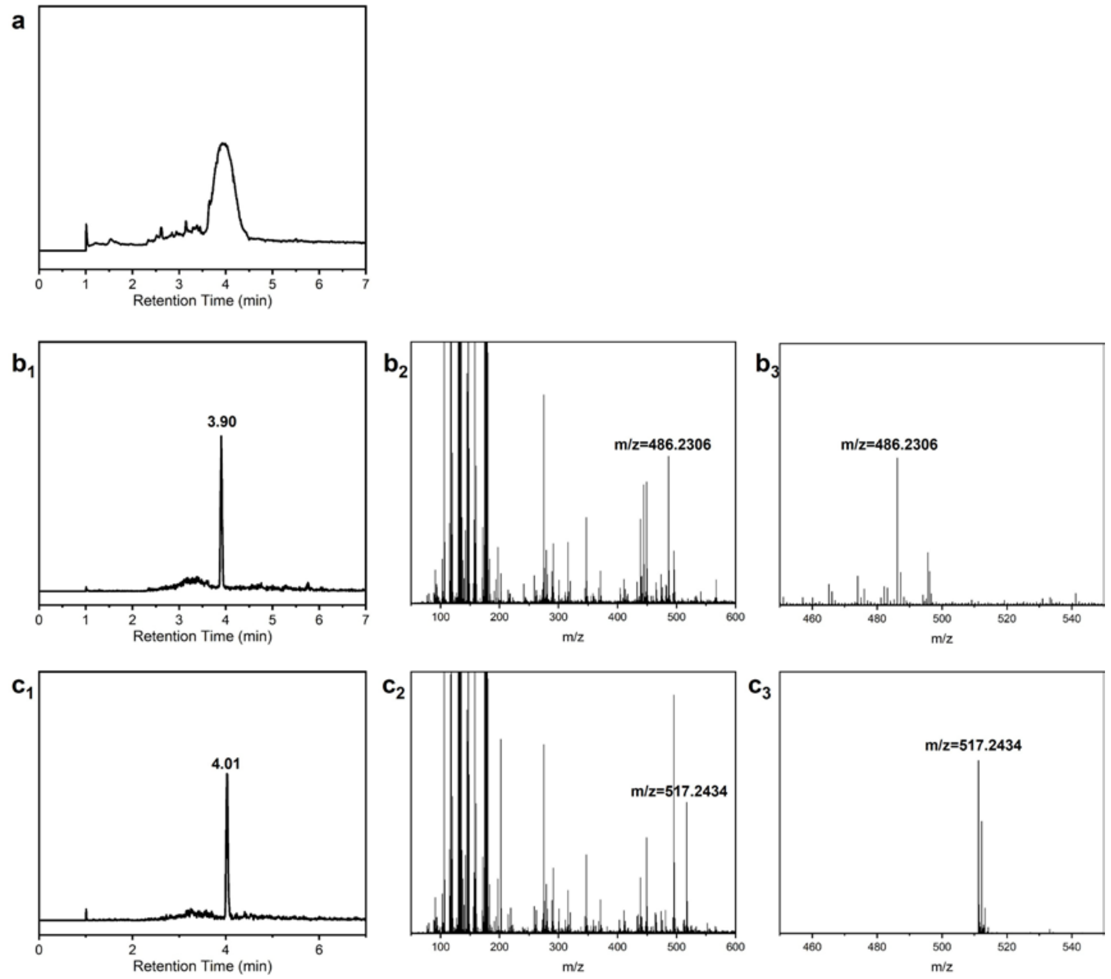

Fig. S15. HPLC-MS of PMP derivatized products from isotope labelling experiments with $^{13}CO_2$ in 10 mM $Ca(OH)_2$ solutions. Total ion chromatogram (a); extracted ion chromatogram (m/z=486.23) (b_1), full (b_2) and zoomed (b_3) mass spectrum at retention time = 3.90 min; and extracted ion chromatogram (m/z=517.24) (c_1), full (c_2) and zoomed mass spectrum at retention time = 4.01 min.

As shown in Fig. S15, signal corresponding to $^{13}C_5$-labeled-ribose and $^{13}C_6$-labeled-glucose could be observed from HPLC-MS of PMP derivatized products from isotope labelling experiments with $^{13}CO_2$ in 10 mM $Ca(OH)_2$ solutions. In the Fig. S15b_1-b_3, retention time of $^{13}C_5$-labeled-ribose is same with standard ribose (Fig. S14a_1) while the peak with m/z=486.2306 could be observed, which is almost same to theoretical molecular weight of $^{13}C_5$-labeled-ribose (m/z=486.2312) In the Figs. S15c_1-c_3, retention time of $^{13}C_6$-labeled-glucose is same with standard ribose (Fig. S14b_1) while the peak with m/z=517.2434 could be observed, which is almost same to theoretical molecular weight of $^{13}C_6$-labeled-glucose (m/z=517.2436).

Table S3. Major formulas in the ordinary differential equations for single bubble dynamics and chemical reactions.

Components	Equations
Bubble radial dynamics	$\left(1-\frac{\dot{R}}{C}\right)R\ddot{R}+\frac{3}{2}\left(1+\frac{\dot{R}}{3C}\right)\dot{R}^2=\left(1+\frac{\dot{R}}{C}\right)\frac{P_i-P_t}{\rho}+\frac{R\dot{P_\iota}}{\rho C}-\frac{4\mu\dot{R}}{\rho R}-\frac{2\sigma}{\rho R}$ $P_i=\frac{N_{tot}k_BT}{V-\frac{4}{3}\pi(R_0/8.86)^3}$
Mass transfer	$\dot{N}_i^d=4\pi R^2D_i\left(\frac{C_{iR}-C_i}{l_{i,diff}}\right); l_{i,diff}=min\left(\sqrt{\frac{RD_i}{\lvert\dot{R}\rvert}},\frac{R}{\pi}\right)$
Heat transfer	$\dot{Q}_{trans}=4\pi R^2\lambda\left(\frac{T_0-T}{l_{th}}\right); l_{th}=min\left(\sqrt{\frac{R\chi}{\lvert\dot{R}\rvert}},\frac{R}{\pi}\right)$
Energy balance	$\dot{E}=-p_i\dot{V}+\dot{Q}+\sum_i h_w\dot{N_\iota^d}$
Chemical reactions	$r_i=r_{f,i}-r_{b,i}$ $r_{f,i}=k_{f,i}[n_A]^a[n_B]^bT^{c_{f,i}}exp\left(-\frac{E_{f,i}}{kT}\right)$ $r_{b,i}=k_{b,i}[n_C]^c[n_D]^dT^{c_{b,i}}exp\left(-\frac{E_{b,i}}{kT}\right)$

To describe the "growth-collapse" process of a single cavitation nucleus, a system of ordinary differential equations was established. This integrates the Keller and Miksis (K-M) equation, mass transfer equation, heat conduction equation, and energy balance equation (Table S3). Such a model facilitates the depiction of changes in physical quantities including radius, number of water molecules inside the bubble, temperature, and pressure. The basis for the calculation of chemical reactions is formed by the variations of these physical quantities over time.

The state variables of the system include: the radius of the bubble, the velocity of the bubble wall, the gas temperature inside the bubble, and the number of water molecules inside the bubble. It is assumed that the initial state of the bubble is physically balanced, i.e., the velocity of the bubble wall is zero, the gas temperature inside the bubble is balanced with the external water temperature, and the partial pressure of water molecules is the saturated steam pressure under the current water temperature. To simplify the calculation process, only inert gas and water vapor in the cavitation nucleus were considered during the physical processes, ignoring the influence of various chemical components.

Table S4. Chemical reactions with the rate coefficients.

Chemical reactions	k_f	c_f	E_f	k_b	c_b	E_b
$H_2O + M \leftrightarrow H\cdot + OH\cdot + M$	1.96E+16	-1.62	59700	2.25E+10	-2	0
$O_2 + M \leftrightarrow 2O\cdot + M$	1.58E+11	-0.5	59472	6170	-0.5	0
$H_2O + O\cdot \leftrightarrow 2OH\cdot$	2210	1.4	8368	210	1.4	200
$OH\cdot + M \leftrightarrow O\cdot + H\cdot + M$	4.66E+11	-0.65	51200	4720000	-1	0
$H\cdot + OH\cdot \leftrightarrow H_2 + O\cdot.$	0.0264	2.65	2245	0.0508	2.67	3166
$H_2O + H\cdot \leftrightarrow H_2 + OH\cdot$	1020	1.51	9370	218	1.51	1726
$O\cdot + HO_2\cdot \leftrightarrow OH\cdot + O_2$	18100000	0	-200	3100000	0.26	26083
$OH\cdot + OH\cdot \leftrightarrow H_2O_2$	0.9	0.9	-3050	1.20E+11	0	22900
$OH\cdot + O\cdot \leftrightarrow H\cdot + O_2$	718000	0.36	-342	1.92E+08	0	8270
$H\cdot + H\cdot \leftrightarrow H_2$	2.45E+08	-1.78	480	4.58E+13	-1.4	52500
$H\cdot + O_2 + M \leftrightarrow HO_2\cdot + M$	2000	0	-500	2.46E+09	0	24300

Considering the high temperature and high particle density environment within the cavitation bubble during the cavitation process, which is fundamental for the generation of free radicals, the Arrhenius equation for the rate of chemical reactions was utilized to depict alterations in the quantities of various particles resulting from chemical reactions (Table S4). Both forward and reverse reactions were taken into account for each specific chemical reaction.

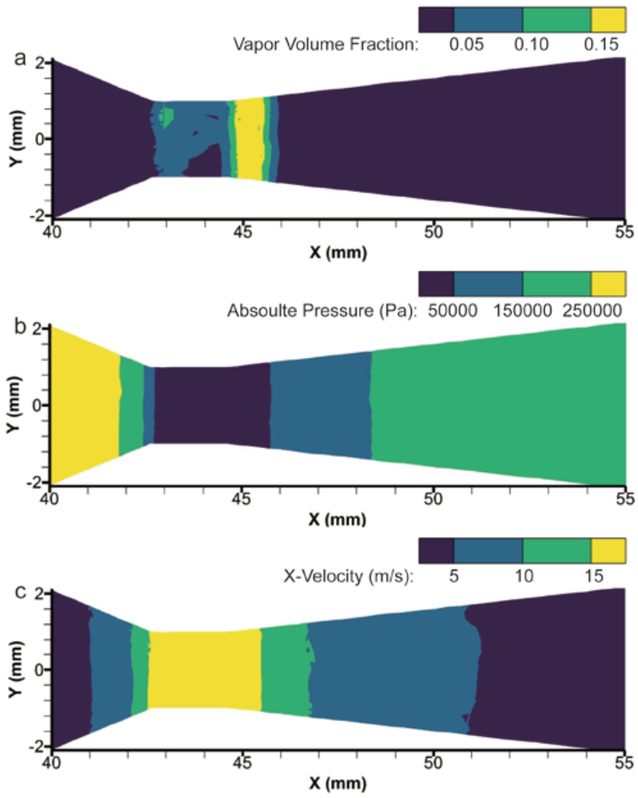

Fig. S16. 2D contour of a Venturi tube for pure water. (**a**) Vapor volume fraction contour. (**b**) Absolute pressure contour. (**c**) Velocity magnitude.

As depicted in Fig. S16, the flow characteristics in pure water are dictated by vapor volume fraction, absolute pressure and velocity magnitude on the vertical plane at Z = 0 mm, with emphasis on the proximity to the throat section. Fig. S16a shows that vapor faction was starting with 0.05 at X = 42.5 mm and then increased along with throat to maximum vapor fraction of 0.27 at X = 45.0 mm, then decreased to 0 at 46.0 mm. It indicates that vapor cavitation occurred in the distance range of 42.5-46.0 mm and a transient collapse of vapor bubbles in the vicinity of X = 46.0 mm. Absolute pressure diminishes from 0.3 MPa at X = 40.0 mm to the saturated vapor pressure in the range of X = 42.5-46.0 mm (Fig. S16b), which promotes cavitation bubbles generation and expansion. Subsequently, absolute pressure was recovered from saturated vapor pressure to 0.2 MPa gradually around from the location of X = 46.0 mm, which is attributed to the transient collapse of vapor bubbles. A rapid escalation is observed in the fluid velocity, with a potential maximum value reaching as high as 22.7 m/s in the range of X = 42.5-46.0 mm (Fig. S16c). The escalation of velocity at throat of Venturi tube caused the diminishment of absolute pressure at the throat, which in line with Bernoulli's equation.

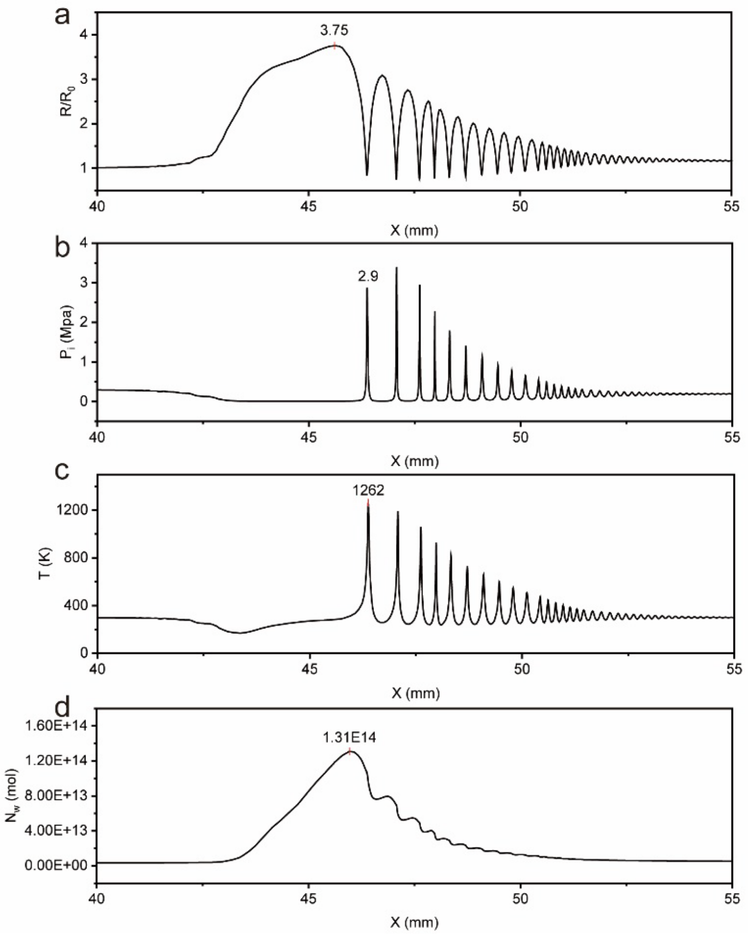

Fig. S17. Results of single bubble dynamics model showing variation in Venturi tube for pure water. (**a**) Normalized radius (R/R_0). (**b**) Collapse pressure. (**c**) Collapse temperature. (**d**) Number of water molecules.

The representative simulation results of single bubble dynamics in pure water, under the conditions of P_{in} = 0.2 MPa and P_{out} = 0.1 MPa, are presented in Fig. S17a-d. As demonstrated in Fig. 17a, the onset of bubble expansion is observed in the vicinity of X = 42.5 mm, until reaching the maximum radius of $3.75R_0$ and then shrinks to $0.84R_0$, occurring near X = 46 mm, which exhibits occurrence, expansion, shrinkage and collapse of cavitation bubble in the cavitation area. As shown in Fig. S17b and c, during the initial moment of collapse, increases in both collapse pressure and temperature are recorded, reaching values of 2.9 MPa and 1262 K around X = 46.0 mm, respectively. In the process of bubble expansion, diffusion of water molecules into the bubble ($N_{w,max}$ = 1.31E-14 mol) was observed (Fig. S17d). Due to the faster kinematics of bubble motion compared to the diffusion kinetics of the water molecules, a majority of the water molecules are trapped within the bubble. Concomitantly, the undiffused water molecules undergo dissociation resulting in the production of free radicals such as OH· and H·. Subsequently, the bubble undergoes periods of inertial oscillations, accompanied by a progressive reduction in energy.

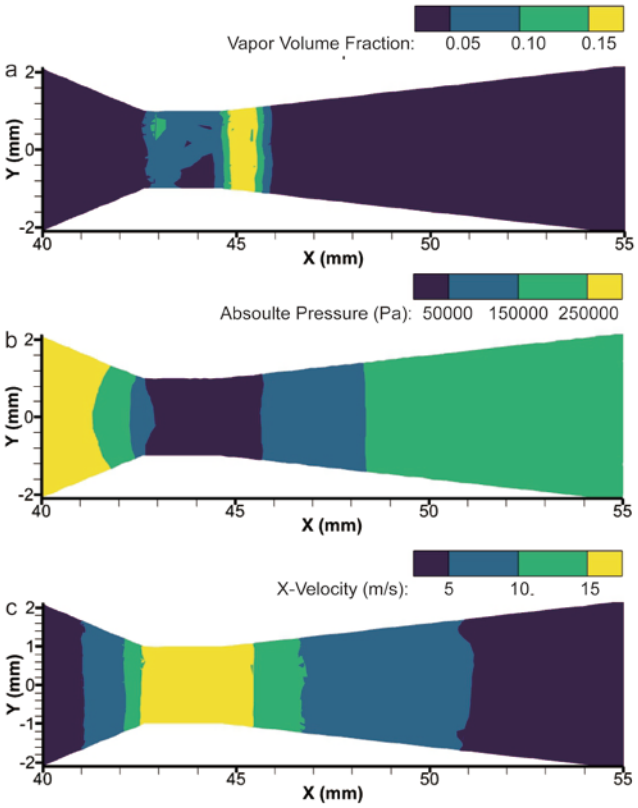

Fig. S18. 2D contour of a Venturi tube for seawater. (**a**) Vapor volume fraction contour. (**b**) Absolute pressure contour. (**c**) Velocity magnitude.

As depicted in Fig. S18, analogous exercises are conducted for CFD simulation in seawater. In this scenario, the vapor volume fraction, absolute pressure, and velocity magnitude exhibit similar values to those in pure water, attributable to the similar saturation vapor pressure in seawater.

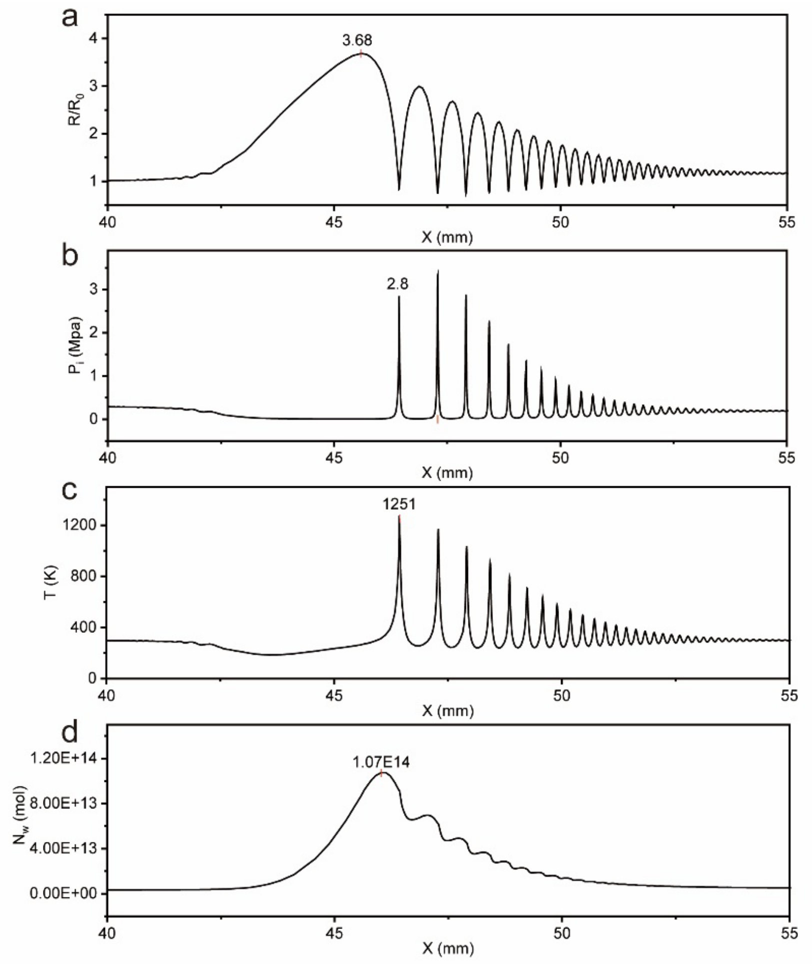

Fig. S19. Results of single bubble dynamics model showing variation in Venturi tube for seawater. (**a**) Normalized radius (R/R_0). (**b**) Collapse pressure. (**c**) Collapse temperature. (**d**) Number of water molecules.

As demonstrated in Fig. S19, similar simulations of single bubble dynamics model are also executed in seawater. Compared to pure water, the collapse pressure and temperature of a single bubble in seawater are similar, valued at 2.8 MPa and 1251.3 K, respectively. This primarily stems from the similar saturation vapor pressure of seawater under the same temperature conditions.

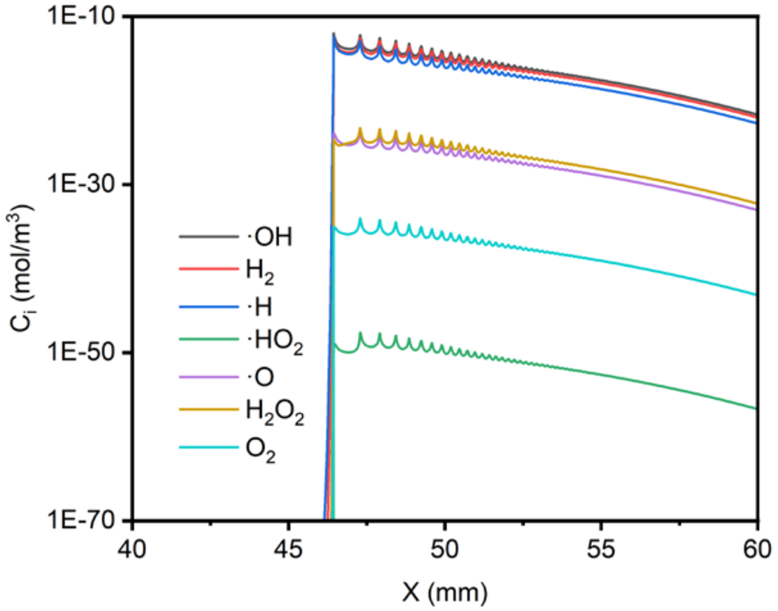

Fig. S20. Production of free radicals in seawater.

Fig. S20 shows the main reactive species during single bubble oscillation in seawater. Reactive species are mainly produced during the initial collapse of a cavitation bubble in the vicinity of X = 46 mm, where the water molecules entrapped in cavitation bubble undergo thermal dissociation under high temperature and pressure conditions. Subsequently, the transformation and diffusion of reactive species result in a downward trend seen in their overall production inside the bubbles. Particularly, within a range of X = 46-52.5 mm, oscillations of limited magnitude in the bubble volume results in fluctuations in the concentration of reactive species. A consistent decrease in the concentration of reactive species is observed when the X position exceeds 52.5 mm, primarily attributed to diffusion. The chemical reactions involved in the collapse process generate ·OH·, H_2, H·, O·, H_2O_2, O_2·, HO_2·, in descending order by the produced amount. Especially, the maximum molecules density for OH· ($C_{i,max}$) is 9.67E-13 mol/m^3, which is similar to the yield in pure water.

Table S5-1. Solvation free energies at M052X/6-31G* level of theory with or without SMD_{DCM} implicit solvent model.

Structure number	$E_{M052X/6\text{-}31G*}$ (a.u.)	$E_{M052X/6\text{-}31G*,SMD(PhCl)}$ (a.u.)	ΔG_{solve} (a.u.)
$H(H_2O)_{25}$	-1910.626463	-1910.747963	-0.121500
$OH(H_2O)_{25}$	-1985.859147	-1985.980013	-0.120866
H_2O	-76.393802	-76.408114	-0.014312
$(H_2O)_{25}$	-1910.136393	-1910.258162	-0.121768
C1-1	-264.956182	-264.974420	-0.018238
TS-1	-265.423066	-265.436465	-0.013399
C1-2	-265.464761	-265.483681	-0.018920
TS-2	-341.834569	-341.863341	-0.028772
C1-3	-266.120139	-266.147723	-0.027583
C1-4	-189.724926	-189.734409	-0.009483
TS-3	-190.197136	-190.203061	-0.005925
C1-5	-190.243332	-190.255860	-0.012528
TS-4	-266.609072	-266.634594	-0.025522
C1-6	-190.895785	-190.910381	-0.014596
C1-7	-114.475292	-114.478360	-0.003068
TS-5	-114.955090	-114.954812	0.000278
C1-8	-115.018817	-115.023694	-0.004877
C2-1	-305.409649	-305.440531	-0.030882
C2-2	-228.990924	-229.005124	-0.014200
C2-3	-228.971062	-228.988204	-0.017142
TS-6	-608.437266	-608.467993	-0.030727
C3-1	-343.506143	-343.525328	-0.019185
C3-2	-419.933204	-419.962459	-0.029255
TS-7	-837.471558	-837.508367	-0.036810
C5-1	-572.521898	-572.561108	-0.039209
C5-2	-572.542106	-572.573090	-0.030984

Table S5-2. Electron energies and Gibbs free energies (298.15 K, 0.1 MPa).

Structure number	Electron energies (a.u.)	Thermal Correction to free energies (a.u.)	Free energies (kcal/mol)
$H(H_2O)_{25}$	-1909.392141	0.531632	-1197902.40
$OH(H_2O)_{25}$	-1984.560777	0.539198	-1245066.29
H_2O	-76.339271	0.003120	-47908.75
$(H_2O)_{25}$	-1908.889763	0.535188	-1197585.09
C1-1	-264.692368	0.012703	-166098.55
TS-1	-265.162746	0.013152	-166390.40
C1-2	-265.203743	0.019125	-166415.84
TS-2	-341.510104	0.036888	-214293.84
C1-3	-265.862898	0.035031	-166824.92
C1-4	-189.531032	0.009590	-118930.56
TS-3	-190.005060	0.009521	-119225.83
C1-5	-190.052425	0.016857	-119255.09
TS-4	-266.355514	0.035533	-167132.43
C1-6	-190.707105	0.030815	-119658.44
C1-7	-114.352034	0.005731	-71753.42
TS-5	-114.831336	0.004292	-72052.99
C1-8	-114.897763	0.010787	-72093.83
C2-1	-305.094092	0.061109	-191428.57
C2-2	-228.740029	0.033802	-143522.34
C2-3	-228.722913	0.033889	-143513.39
TS-6	-607.790813	0.091290	-381354.60
C3-1	-343.126353	0.063520	-215285.32
C3-2	-419.486561	0.091923	-263190.57
TS-7	-836.566531	0.152946	-524878.65
C5-1	-571.887403	0.124858	-358809.12
C5-2	-571.903380	0.126236	-358813.12

Table S5-3. Electron energies and Gibbs free energies (1262 K 29 atm).

Structure number	Electron energies (a.u.)	Thermal Correction to free energies (a.u.)	Free energies (kcal/mol)
$H(H_2O)_{25}$	-1909.392141	-0.205817	-1198363.86
$OH(H_2O)_{25}$	-1984.560777	-0.210733	-1245535.58
H_2O	-76.339271	-0.064398	-47949.82
$(H_2O)_{25}$	-1908.889763	-0.193970	-1198041.35
C1-1	-264.692368	-0.098759	-166167.20
TS-1	-265.162746	-0.108815	-166465.64
C1-2	-265.203743	-0.106688	-166493.49
TS-2	-341.510104	-0.115719	-214388.30
C1-3	-265.862898	-0.086647	-166899.98
C1-4	-189.531032	-0.087445	-118990.15
TS-3	-190.005060	-0.098632	-119292.40
C1-5	-190.052425	-0.093200	-119322.85
TS-4	-266.355514	-0.101418	-167217.07
C1-6	-190.707105	-0.069545	-119720.12
C1-7	-114.352034	-0.075463	-71803.07
TS-5	-114.831336	-0.092273	-72112.29
C1-8	-114.897763	-0.077440	-72147.90
C2-1	-305.094092	-0.084436	-191518.61
C2-2	-228.740029	-0.087718	-143597.30
C2-3	-228.722913	-0.088979	-143589.20
TS-6	-607.790813	-0.122358	-381487.37
C3-1	-343.126353	-0.093775	-215382.73
C3-2	-419.486561	-0.083891	-263299.60
TS-7	-836.566531	-0.117964	-525047.36
C5-1	-571.887403	-0.093326	-358944.74
C5-2	-571.903380	-0.088584	-358946.63

Table. S5-4 Fully-optimized geometries.

$H(H_2O)_{25}$
0 2
O -0.000244 -1.022087 -0.141517
H 0.911315 -1.417759 -0.082812
H -0.723048 -2.279101 -0.670660
O 0.954664 -1.651197 2.636095
H 0.592782 -0.760825 2.509944
H 1.887215 -1.561452 2.353799
O 2.222605 -2.483646 -0.067962
H 2.781151 -2.319719 -0.866307
H 2.811090 -2.220279 0.721807
O 2.659264 0.627501 0.867540
H 1.769593 0.661532 1.265953
H 2.475131 0.507384 -0.810520
O 2.615927 3.411241 0.718449
H 2.727244 2.451447 0.881792
H 0.977840 3.649627 1.163290
O -0.291102 0.594528 -2.289587
H -0.759480 1.308789 -1.791274
H -0.012419 -0.376993 -0.887211
O -2.551015 -0.957708 -2.720850
H -2.770277 -1.139917 -3.645964
H -1.002676 -0.008731 -2.590368
O -1.096964 -3.193881 -0.938409
H -1.922905 -3.363112 -0.357066
H -0.340762 -3.875772 -0.657304
O 0.415605 1.194202 2.498514
H -1.162577 0.959032 3.352486
H 1.094363 1.379135 3.165256
O -3.748848 1.243625 -1.928274
H -3.017047 -0.095860 -2.518832
H -3.934521 0.860933 -1.043183
O 3.650646 -1.508788 1.770960
H 3.949661 -1.779553 2.647779
H 3.113400 -0.166495 1.324301
O 2.485711 3.213643 -2.046869
H 3.277026 3.509465 -2.517210
H 2.613040 3.452988 -0.260787
O 3.876130 -1.790717 -2.099342
H 3.402171 -0.934591 -2.142633
H 4.553070 -1.637055 -1.398499
O 5.569508 -1.483298 0.013284
H 5.930614 -2.379574 0.044470
H 4.866656 -1.488633 0.736306
O -2.049829 -1.209625 1.645828
H -1.237745 -1.116341 1.102633
H -2.383453 -2.107070 1.457744
O -3.177233 -3.446994 0.500044
H -3.825629 -2.944386 -0.080609
H -3.530534 -4.340498 0.613692
O 0.207866 4.567819 -1.393824
H 1.006266 4.081074 -1.691016
H -0.087161 5.039320 -2.184998
O 2.434199 0.539483 -1.803932
H 1.480876 0.476662 -2.021744
H 2.535740 2.224954 -2.020778
O 0.789489 -4.643719 -0.234352
H 1.122930 -5.124167 -1.005986
H 1.432130 -3.881221 -0.115714
O -4.608471 -1.953273 -1.080510
H -4.709737 -1.147935 -0.540541
H -3.888290 -1.715058 -1.701529
O -2.118712 0.806085 3.495048
H -2.549249 1.899858 2.270423
H -2.254890 -0.004673 2.964348
O -1.565017 2.580415 -0.970649
H -0.950773 3.349014 -1.068697
H -3.010461 1.859136 -1.740959
O 0.043681 3.619162 1.495874
H 0.214517 2.089341 2.105886
H 0.034102 4.226354 2.249371
O -4.161090 0.215302 0.619780
H -3.364959 -0.290001 0.904028
H -3.970298 1.114492 0.953182
O -2.815544 2.430850 1.475278
H -3.057368 3.300751 1.821870
H -1.819238 2.536726 -0.023889
H 0.795762 1.947369 -0.338212

$OH(H_2O)_{25}$
0 2
O 0.244213 1.582179 -0.187368
H -0.654372 2.000396 -0.144966
H 1.140587 2.648440 -0.603551
O -0.866880 0.933785 2.910878
H -1.077817 -0.016052 2.935941
H -1.670930 1.335518 2.541608

O -1.920157 3.115316 -0.097858
H -2.406178 2.825023 -0.920497
H -2.484771 2.831259 0.648092
O -2.422837 -0.142596 0.465359
H -1.512746 -0.254566 0.784141
H -2.268985 -0.222388 -1.009063
O -3.479934 -2.491039 0.472269
H -3.111245 -1.548452 0.516730
H -1.974726 -3.088322 0.680805
O 0.575951 -0.442253 -1.926479
H 0.959544 -1.292676 -1.601641
H 0.216487 0.776038 -0.757761
O 2.899468 0.908528 -2.539968
H 3.067606 1.269545 -3.421542
H 1.334342 0.071485 -2.275916
O 1.693081 3.521974 -0.757910
H 2.429014 3.489852 -0.051931
H 1.007242 4.261451 -0.510353
O -1.185644 -1.881151 3.138785
H 0.573560 -1.480282 3.278163
H -1.463115 -2.334393 3.947234
O 3.786184 -1.518475 -2.107770
H 3.280454 -0.014153 -2.550790
H 3.788779 -1.250920 -1.158844
O -3.400407 1.719708 1.702547
H -3.857364 1.388755 2.486733
H -2.993888 0.860904 1.174240
O -2.872907 -2.838834 -2.227413
H -3.419164 -2.968092 -3.014802
H -3.497504 -2.672625 -0.486056
O -3.246120 2.186206 -2.189043
H -2.859843 1.281998 -2.234125
H -4.030726 2.088772 -1.604744
O -5.195155 2.267962 -0.272911
H -5.212148 3.235382 -0.277382
H -4.599562 2.059234 0.485106
O 2.025036 0.958222 1.785833
H 1.240399 1.123996 1.220244
H 2.553138 1.776860 1.719127
O 3.528110 3.128438 0.965267
H 4.110294 2.513044 0.426118
H 4.096301 3.838088 1.296079
O -0.707504 -4.338798 -1.728089
H -1.511129 -3.831719 -2.006700
H -0.883376 -5.261921 -1.954206
O -2.158123 -0.311828 -2.035674
H -1.185941 -0.326110 -2.154757
H -2.612972 -1.872179 -2.237263
O -0.147349 5.126893 -0.161245
H -0.362956 5.647813 -0.948876
H -0.875743 4.458434 -0.102890
O 4.782224 1.406698 -0.542804
H 4.664591 0.556423 -0.082961
H 4.130670 1.349314 -1.274484
O 1.546750 -1.377415 3.255517
H 1.949925 -2.554432 2.123823
H 1.693787 -0.530726 2.788528
O 1.549036 -2.889681 -1.269545
H 0.798844 -3.485699 -1.474956
H 3.022584 -2.126561 -2.118219
O -1.022140 -3.370842 0.809000
H -1.087723 -2.585626 2.471560
H -0.815741 -3.882777 -0.000773
O 3.591003 -0.886570 0.526025
H 2.943326 -0.228043 0.868171
H 3.252224 -1.747676 0.844342
O 2.253300 -3.170608 1.404538
H 2.859880 -3.789326 1.833477
H 1.736105 -3.007056 -0.314913
O 0.364482 -1.111703 0.797474
H -0.128476 -1.988176 0.709374

H_2O

0 1
O 0.000000 0.000000 0.121054
H 0.000000 0.752864 -0.484218
H 0.000000 -0.752864 -0.484218

$(H_2O)_{25}$

0 1
O -0.612940 1.019390 -0.255411
H -1.611087 1.076268 -0.310047
H -0.247862 2.441380 -0.405439
O -1.907063 1.209510 2.466404
H -1.351363 0.462078 2.755298
H -2.697815 0.770022 2.100482
O -3.205961 1.451169 -0.398456
H -3.509280 1.009427 -1.230886
H -3.660570 0.932654 0.343622
O -2.175617 -1.467725 0.690152

H -1.457596 -0.831803 0.837751
H -1.977440 -1.568736 -0.987984
O -0.759604 -3.769314 1.083237
H -1.423376 -3.048018 1.024092
H 0.394280 -2.419944 0.752131
O 0.377946 -0.120606 -2.505993
H 1.120650 -0.722488 -2.261143
H -0.291247 0.495839 -1.027796
O 1.961728 2.126298 -2.359293
H 1.929532 2.794499 -3.059528
H 0.829762 0.742559 -2.620943
O -0.160844 3.477438 -0.427584
H 0.430737 3.729908 0.361635
H -1.143707 3.793281 -0.259516
O -0.410292 -1.119885 3.100485
H 1.535558 -1.282588 3.633913
H -1.090779 -1.804339 3.017716
O 4.309445 0.768794 -2.427595
H 2.807706 1.639272 -2.520971
H 4.287136 0.854107 -1.458430
O -4.178323 -0.127432 1.345767
H -4.577989 -0.074779 2.222994
H -3.024731 -0.982211 1.035494
O -0.113714 -3.803318 -1.594439
H -0.353321 -4.497260 -2.223667
H -0.624190 -4.014997 0.147118
O -4.054217 -0.072525 -2.452714
H -3.326091 -0.720761 -2.382635
H -4.735619 -0.414969 -1.824819
O -5.830072 -0.801139 -0.539601
H -6.433270 -0.047153 -0.592029
H -5.229578 -0.566969 0.236982
O 1.190323 1.148149 1.846429
H 0.428801 0.984412 1.252128
H 1.250900 2.122131 1.907823
O 1.508263 3.857892 1.460310
H 2.325066 3.704923 0.901083
H 1.622032 4.705897 1.911866
O 2.345082 -3.770757 -0.406160
H 1.450480 -3.803990 -0.815076
H 2.839819 -4.481977 -0.837012
O -1.816858 -1.713833 -1.956929
H -1.145198 -1.047682 -2.204631
H -0.731169 -3.054488 -1.782530
O -2.579977 4.001395 -0.092225
H -2.894526 4.462296 -0.883631
H -2.917428 3.070131 -0.198690
O 3.430758 3.176732 -0.177648
H 3.690365 2.303531 0.167620
H 2.854073 2.932203 -0.930961
O 2.378135 -0.893376 3.348225
H 3.241420 -1.823010 2.126266
H 2.067269 -0.111449 2.848565
O 2.651240 -1.391560 -1.792395
H 2.628125 -2.255586 -1.328748
H 3.972678 -0.136767 -2.546797
O 0.729179 -1.507501 0.609348
H 0.051056 -1.161272 2.233309
H 1.675478 -1.555649 0.805818
O 3.285572 0.422731 0.234386
H 2.497483 0.696095 0.759986
H 3.595067 -0.404804 0.671649
O 3.752734 -2.028723 1.310828
H 3.263453 -2.724377 0.832993
H 2.746947 -0.739277 -1.067444

C1-1

0 1
O 0.625994 1.173501 -0.000030
C 0.042184 0.120751 -0.000007
O -1.280628 0.042085 0.000059
H -1.573193 -0.884398 -0.000098
O 0.621332 -1.075848 -0.000096
H 1.586510 -0.958016 0.000678

TS-1

0 2
O -0.985561 -0.869776 0.024327
C 0.031014 -0.027045 -0.132423
O -0.289487 1.254510 0.057040
H 0.510117 1.797663 -0.042892
O 1.205754 -0.453066 -0.124814
H 1.678054 -0.707704 1.066617
H -1.819904 -0.381031 0.118387

C1-2

0 2
O 1.186156 -0.575046 0.088510
C 0.008976 -0.016128 -0.309087
O -0.096116 1.282316 0.114700

H -0.923664 1.647953 -0.233838
O -1.060748 -0.812210 -0.044256
H -1.265846 -0.766146 0.909164
H 1.901318 0.054477 -0.092431

TS-2

0 2
O -0.153789 -0.912865 0.974379
C -0.338528 -0.004537 -0.045356
O -0.527793 1.256722 0.482014
H -0.647373 1.880234 -0.250920
O -1.431786 -0.342423 -0.814141
H -1.272711 -1.216908 -1.201908
H 0.799575 -0.937090 1.154547
H 0.626832 -0.013594 -0.688758
O 2.155915 -0.069563 -0.464698
H 2.184461 0.859615 -0.161249

C1-3

0 1
O 1.287306 -0.187139 -0.175943
C 0.000143 -0.000207 0.316667
O -0.806060 -1.020679 -0.175818
H -1.649414 -0.992539 0.299078
O -0.481534 1.207984 -0.175577
H -0.035588 1.924823 0.298590
H 1.685267 -0.931429 0.298943
H 0.001181 -0.000938 1.422089

C1-4

0 1
O 1.156171 0.087048 0.000000
C 0.000000 0.420206 0.000000
O -1.026582 -0.419100 0.000000
H -0.686767 -1.333516 0.000000
H -0.349944 1.468692 0.000000

TS-3

0 2
O -1.089110 -0.140173 -0.157331
C -0.014266 0.463401 0.004472
O 1.161549 -0.156865 0.029695
H 1.032468 -1.095775 -0.194967
H 0.055178 1.528205 0.266240
H -1.581561 -0.836533 0.922983

C1-5

0 2
O -1.083126 -0.324640 -0.063055
C -0.004389 0.492927 0.160522
O 1.184687 -0.128614 -0.076126
H 1.150938 -1.014088 0.319154
H -0.046192 1.500304 -0.274968
H -1.890904 0.182258 0.106129

TS-4

0 2
O -0.771933 1.201006 -0.173431
C -0.525122 0.005250 0.490363
O -0.927695 -1.130395 -0.197693
H -0.318678 -1.263809 -0.940400
H 0.608062 -0.085692 0.701570
H -0.230444 1.223832 -0.977993
H -1.047902 0.036023 1.455576
O 1.969218 -0.163530 -0.006357
H 1.982983 0.801494 -0.161076

C1-6

0 1
O 1.064038 -0.379523 0.000000
C 0.002649 0.539160 0.000000
O -1.194396 -0.193519 0.000000
H -0.947457 -1.129102 0.000002
H 0.038471 1.183344 0.897509
H 1.897488 0.111786 0.000000
H 0.038470 1.183345 -0.897508

C1-7

0 1
O 0.000000 0.000000 0.675574
C 0.000000 0.000000 -0.528864
H 0.000000 0.943360 -1.115703
H 0.000000 -0.943360 -1.115703

TS-5

0 2
O -0.576336 -0.001536 -0.195875
C 0.633725 0.000416 0.045195
H 1.190710 0.946617 0.169297
H 1.191552 -0.943889 0.179543
H -1.573921 0.007064 0.946987

C1-8

0 2
O -0.053165 -0.687168 0.000000
C -0.053165 0.682832 0.000000

H -0.056722 1.210732 0.956694
H -0.056722 1.210732 -0.956694
H 0.857758 -1.021115 0.000000

C2-1

0 1
O 1.942962 0.104881 0.047131
C 0.781607 -0.632188 -0.255571
H 0.682558 -0.664296 -1.351270
H 2.075402 0.067050 1.004012
H 0.837101 -1.668848 0.114279
C -0.472459 0.010226 0.300994
H -0.407188 0.084091 1.402310
O -1.545092 -0.818250 -0.063756
H -2.328663 -0.533739 0.426845
O -0.584453 1.294740 -0.251390
H -1.221433 1.796545 0.275395

C2-2

0 1
O -1.706127 -0.216160 -0.036100
C -0.512935 0.526889 -0.036802
H -0.497283 1.147399 -0.947825
H -1.772623 -0.673782 0.813104
H -0.421243 1.211806 0.823521
C 0.709400 -0.353236 -0.090237
H 0.505633 -1.424824 -0.325257
O 1.831968 0.053346 0.085936

C2-3

0 1
O -1.797402 0.219367 -0.000229
C -0.554931 -0.369041 -0.000159
H -2.466562 -0.476508 0.002211
H -0.513462 -1.461625 -0.000381
C 0.554900 0.369067 0.000017
H 0.513355 1.461665 0.000281
O 1.797441 -0.219400 0.000031
H 2.466544 0.476576 0.000320

TS-6

0 1
O -3.255275 -0.296222 -1.095821
C -2.529631 -0.434328 0.042825
H -4.069657 0.186074 -0.896683
H -3.019702 -0.259847 1.004654
C -1.275289 -0.957944 -0.009403
H -0.808156 -1.173789 -0.978998
O -0.573358 -1.166912 1.091608
C -1.262983 1.401596 -0.035134
H -0.968113 1.274637 -1.089068
H -2.255708 1.840010 0.136627
O -0.386375 1.515654 0.867911
H 0.405647 -1.192046 0.834277
H 0.711653 1.217204 0.437420
O 1.762847 1.083314 -0.080573
C 2.288936 -0.073264 -0.157510
O 1.811432 -1.145922 0.233582
O 3.495665 -0.057748 -0.733574
H 3.838347 -0.965900 -0.757971

C3-1

0 1
O 1.864021 -0.623617 -0.143541
C 0.598986 -0.219255 0.324313
H 2.540450 -0.285929 0.459403
H 0.505662 -0.335168 1.421063
C 0.375131 1.249452 0.025183
H 1.298337 1.870380 -0.030038
O -0.717316 1.739891 -0.145988
C -0.470469 -1.088126 -0.331242
H -0.461767 -0.910042 -1.421453
H -0.206613 -2.140419 -0.159327
O -1.741363 -0.857627 0.231240
H -1.940684 0.079582 0.087137

C3-2

0 1
O 0.336979 2.015387 -0.143526
C -0.092252 0.715509 -0.483891
H 1.053087 2.262429 -0.743963
H -0.357148 0.644135 -1.553643
C 1.015877 -0.310274 -0.236666
O 0.679224 -1.566470 -0.758906
C -1.334693 0.449466 0.348770
H -1.069816 0.534874 1.414440
H -2.083422 1.224780 0.116588
O -1.819259 -0.844308 0.038945
H -2.466151 -1.100730 0.709371
H -0.233975 -1.743192 -0.468895
H 1.924674 0.015678 -0.772356
O 1.259464 -0.362171 1.144353
H 2.047893 -0.905685 1.282251

TS-7
0 1
O 2.223254 -1.363226 1.545409
C 2.134431 -1.176320 0.158636
H 1.400988 -1.792194 1.829266
H 2.166755 -2.147184 -0.380371
C 0.756356 -0.670190 -0.237736
O -0.194538 -1.235556 0.386397
C 3.341406 -0.366957 -0.279772
H 3.491267 0.481536 0.408841
H 4.231338 -1.009713 -0.216508
O 3.123802 0.079883 -1.604716
H 3.969498 0.362744 -1.980953
H 0.612075 -0.336098 -1.276990
C 1.012185 1.445624 0.395471
C -0.345886 1.500108 0.519384
O 1.596005 2.110998 -0.656932
H 1.624200 1.175499 1.265007
O -0.971677 1.017403 1.559532
H -0.946224 1.861538 -0.328256
H 2.195621 1.504074 -1.141601
H -1.941016 0.815089 1.284255
O -3.242672 0.509893 0.647333
C -3.250035 -0.260034 -0.322457
O -2.285339 -0.977716 -0.745463
O -4.391882 -0.353962 -1.006971
H -4.280483 -0.987202 -1.734658
H -1.270379 -1.063204 -0.125896

C5-1
0 1
O 1.215456 0.907320 1.528985
C 1.271645 -0.176420 0.620433
H 0.715918 0.629186 2.309959
H 1.699425 -1.071115 1.106527
C -0.118960 -0.595043 0.109894
O -0.685029 -1.628421 0.884989
C 2.219840 0.222288 -0.498909
H 1.778270 1.039986 -1.090735
H 3.150976 0.594105 -0.037901
O 2.450197 -0.921826 -1.291968
H 2.884548 -0.639508 -2.106470
H 0.007710 -1.004274 -0.904327
C -1.094329 0.604965 0.074009
C -2.302061 0.218242 -0.739861
O -0.534650 1.755522 -0.510421
H -1.426140 0.790866 1.112115
O -3.156113 -0.539756 -0.351362
H -2.336717 0.666602 -1.759798
H 0.146610 2.065780 0.106186
H -0.796298 -1.298522 1.789265

C5-2
0 1
O -1.110944 1.966863 -0.404989
C -1.012730 0.561482 -0.359083
H -0.217237 2.338587 -0.341721
H -0.694796 0.142445 -1.334410
C 0.008483 0.130562 0.716267
O -0.070202 -1.241701 1.023050
C -2.406056 0.029563 -0.046288
H -2.667722 0.306271 0.991955
H -3.111635 0.543131 -0.715379
O -2.530487 -1.356023 -0.269785
H -1.872581 -1.778138 0.302987
H -0.234654 0.695207 1.631133
C 1.397250 0.575404 0.277624
C 2.420628 -0.486031 -0.079501
O 1.650401 1.759338 0.181012
O 1.850191 -1.536778 -0.831879
H 3.256524 0.001819 -0.601644
H 0.350760 -1.727207 0.289085
H 2.799618 -0.916558 0.860520
H 1.634599 -1.205036 -1.715907

References

1. Garcia, A. D. Resolution and 1uantification of carbohydrates by enantioselective comprehensive two-dimensional gas chromatography. *Talanta* 271, 125728-125740 (2024).

2. Schnerr, G. and Sauer, J. Physical and numerical modeling of unsteady cavitation dynamics. *In: 4th Int Conf Multiph Flow* (2001).

3. Dastane, G. G. et al. Single and multiphase CFD simulations for designing cavitating venturi. *Chem Eng Res Des* **149**, 1-12 (2019).

4. Pandit, A. V. et al. Estimation of chemical and physical effects of cavitation by analysis of cavitating single bubble dynamics, *Ultrason Sonochem* **77**, 105677 (2021).

5. Pawar, S. *et al.* Sonochemical effect induced by hydrodynamic cavitation: comparison of Venturi/Orifice flow geometries, *AlChE J* **63**, 4705–4716 (2017).

6. Yasui, K. et al. Theoretical study of single-bubble sonochemistry, *J Chem Phys* **122**, 224706 (2005).

7. Yasui, K. et al. Optimum bubble temperature for the sonochemical production of oxidants, *Ultrasonics* **42**, 579-584 (2004).

8. Zhao, Y. et al. Exchange-correlation functional with broad accuracy for metallic and nonmetallic compounds, kinetics, and noncovalent interactions. *J Chem Phys* **123**, 161103 (2005).

9. Grimme, S. et al. Effect of the damping function in dispersion corrected density functional theory. *J Comput Chem* **32**, 1456-1465 (2011).

10. Weigend, F. and Ahlrichs, R. Balanced vasis sets of split valence, triple zeta valence and quadruple zeta valence quality for H to Rn: design and assessment of accuracy. *Phys Chem Chem Phys* **7**, 3297-3305 (2005).

11. Kesharwani, M. et al. Frequency and zero-point vibrational energy scale factors for double-hybrid density functionals (and other selected methods): can anharmonic force fields be avoided. *J Phys Chem A* **119**, 1701-1714 (2015).

12. Grimme, S. Supramolecular binding thermodynamics by dispersion-corrected density functional theory. *Chem Eur J* **18**, 9955-9964 (2012).

13. Lu, T.; Chen, Q. Shermo: A general code for calculating molecular thermochemistry properties. *Comput Theor Chem* **1200**, 113249 (2021).

14. Santra, G. et al. Minimally empirical double-hybrid functionals trained against the GMTKN55 database: revDSD-PBEP86-D4, revDOD-PBE-D4, and DOD-SCAN-D4. *J Phys Chem* A **123**, 5129-5143 (2019).

15. Marenich, A. V. et al. Universal solvation model based on solute electron density and on a continuum model of the solvent defined by the bulk dielectric constant and atomic surface tensions. *J Phys Chem B.* **113**, 6378-6396 (2009).
